# The diversity of the glycan shield of sarbecoviruses related to SARS-CoV-2

**DOI:** 10.1016/j.celrep.2023.112307

**Published:** 2023-03-15

**Authors:** Joel D. Allen, Dylan P. Ivory, Sophie Ge Song, Wan-ting He, Tazio Capozzola, Peter Yong, Dennis R. Burton, Raiees Andrabi, Max Crispin

**Affiliations:** 1 School of Biological Sciences, University of Southampton, Southampton SO17 1BJ, UK; 2 Department of Immunology and Microbiology, The Scripps Research Institute, La Jolla, CA 13 92037, USA; 3 IAVI Neutralizing Antibody Center, The Scripps Research Institute, La Jolla, CA 92037, USA; 4 Consortium for HIV/AIDS Vaccine Development (CHAVD), The Scripps Research Institute, La Jolla, CA 92037, USA; 5 Ragon Institute of Massachusetts General Hospital, Massachusetts Institute of Technology, and Harvard University, Cambridge, MA 02139, USA

**Keywords:** SARS-CoV-2, pan-coronavirus, N-linked glycosylation, glycan shielding

## Abstract

Animal reservoirs of sarbecoviruses represent a significant risk of emergent pandemics, as evidenced by the severe acute respiratory syndrome coronavirus 2 (SARS-CoV-2) pandemic. Vaccines remain successful at limiting severe disease and death, but the potential for further coronavirus zoonosis motivates the search for pan-coronavirus vaccines. This necessitates a better understanding of the glycan shields of coronaviruses, which can occlude potential antibody epitopes on spike glycoproteins. Here, we compare the structure of 12 sarbecovirus glycan shields. Of the 22 N-linked glycan attachment sites present on SARS-CoV-2, 15 are shared by all 12 sarbecoviruses. However, there are significant differences in the processing state at glycan sites in the N-terminal domain, such as N165. Conversely, glycosylation sites in the S2 domain are highly conserved and contain a low abundance of oligomannose-type glycans, suggesting a low glycan shield density. The S2 domain may therefore provide a more attractive target for immunogen design efforts aiming to generate a pan-coronavirus antibody response.

## Highlights

• N-linked glycans are prevalent across SARS-CoV-2-like sarbecoviruses
• 3D maps of sarbecovirus glycan shields demonstrate localized changes in structure
• Key regions of conservation include the C-terminal S2 glycan sites
• SARS-CoV-2 lacks the conserved N370 glycan, which influences viral infectivity

## Graphical abstract

**Figure:**
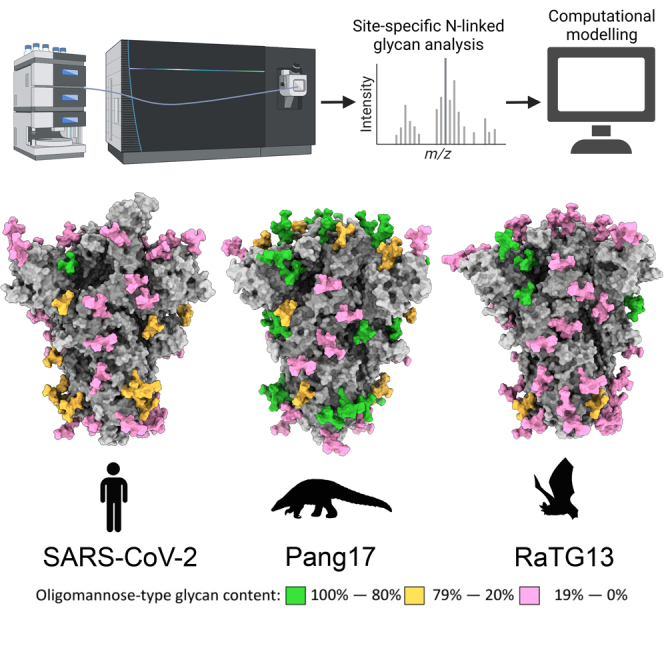

## Teaser

Allen et al. determine the glycosylation of several animal sarbecovirus spike proteins, which have shared receptor usage and high sequence similarity to SARS-CoV-2. This study provides insights into regions of the glycan shield of the S protein that are conserved and informs immunogen design efforts toward a pan-coronavirus immunogen.

## Introduction

For many years, coronaviruses have been considered a significant threat to public health because of their abundance in animal reservoirs and the severity of disease when zoonosis occurs.^1^ Outbreaks occurred in 2003 with the severe acute respiratory syndrome coronavirus 1 (SARS-CoV-1) epidemic in Hong Kong^2^ and in 2010 with the endemic spread of Middle Eastern respiratory syndrome CoVs (MERS-CoV).^3^ CoVs are divided into four genera: alpha, beta, gamma and delta, of which SARS-CoV-2, MERS-CoV, and SARS-CoV-1 belong to the betacoronavirus genera. Betacoronaviruses can be further classified as a sarbecovirus, merbecovirus, embecovirus, or nobecovirus, with SARS-CoV-1 and SARS-CoV-2 classified as sarbecoviruses. Sarbecoviruses can be further grouped into clades, with clade 1a including SARS-CoV-1 and clade 1b including SARS-CoV-2. The most severe pandemic resulting from CoV zoonosis occurred in 2019, when SARS-CoV-2 spread across the globe; as of July 2022, it has resulted in millions of deaths and over half a billion infections worldwide.^4^ The rapid development and deployment of vaccines has proven to be the most resilient measure in minimizing severe disease and death as lockdowns ease. All of the widely used SARS-CoV-2 vaccines are based around the spike (S) glycoprotein.

The CoV S protein mediates receptor binding, enabling the virus to enter host cells. Following translation, the S protein consists of a single 200-kDa polypeptide chain of over 1,200 amino acids, separated into the N-terminal domain (NTD), the receptor binding domain (RBD), fusion peptide (FP), heptad repeat 1 and 2 (HR1/2), and the transmembrane C-terminal domain.^5^ During secretion, the RBD and NTD are separated from the C-terminal elements by proteolytic cleavage; in the case of SARS-CoV-2 this is achieved through the action of the host protease, furin.^6^ The mature S protein located on the surface of virions consists of a trimer of heterodimers of S1 (containing the NTD and RBD) and S2.

In addition to proteolytic cleavage and maturation, the S protein undergoes extensive post-translational modifications as it progresses through the secretory system. The most abundant post-translational modification is N-linked glycosylation, with approximately one-third the mass of the S protein consisting of N-linked glycans.^7^^,^^8^ Glycans are critical for correct folding of the SARS-CoV-2 S protein, and removal of N-linked glycan sites can result in a reduction or loss of ACE2 binding.^9^ Furthermore, the precise processing state of N-linked glycans is influenced by the surrounding glycan and protein architecture. Thus, the viral genome exerts some control over the processing state.^10^ While N-linked glycans can contribute to neutralizing antibody epitopes, particularly in HIV,^11^ their main effect as large, immunologically “self” structures is to occlude the underlying protein surface. This means that changes in the glycan shield, with respect to the position of an N-linked glycan site and the processing state of the attached glycan, can modulate viral infectivity and hamper vaccine design efforts.^12^^,^^13^ Conversely, the presence of underprocessed glycans on viral glycoprotein immunogens, particularly of the oligomannose type, can enhance the interaction with the innate immune system and assist trafficking to germinal centers.^14^ Therefore, research into viral biology and vaccine design efforts benefit from intricate knowledge of the viral glycan shield. Differences in the glycan shield can indicate changes in the protein architecture and, therefore, a changing antigenic surface. For this reason, it is important to understand the presentation and processing of the N-linked glycans on viral S glycoproteins.

When preparing for future pandemics, it is important to note that bats are known reservoirs of SARS-like CoVes.^15^ Viruses isolated from *Rhinolophus sinicus*, such as WIV-1-CoV and RsSHC014-CoV, have been shown to recognize human ACE2 and replicate efficiently in human primary airway epithelial cells,^16^^,^^17^ highlighting the threat to human health that bat CoVs present.^18^ Additionally, bat sarbecovirus RS4081, which cannot bind human ACE2, has been shown to be able to replicate in human kidney and liver cells.^19^ Other sarbecoviruses have demonstrated broad ACE2 recognition, with BtKY72, isolated in Kenya, demonstrating binding to ACE2 from *Rhinolophus affinis*, which are bats located in Asia.^20^ As well as sharing functional properties with SARS-CoV-2, several sarbecoviruses have been described previously that possess remarkable sequence similarity. Notable examples include RaTG13, found in *R. affinis*,^21^ and RmYN02 ^22^ and pang17-CoV,^23^ which are more than 90% conserved with SARS-CoV-2. Additionally, the RBD of BM4831, isolated in Bulgaria, has a higher similarity to the SARS-CoV-1 RBD than any sarbecovirus isolated from Chinese bats.^24^ The capacity of sarbecoviruses to recognize human ACE2 and infect human cells, combined with their high sequence similarity to SARS-CoV-2, underscores their pandemic potential.

The combination of factors outlined above means that a pan-CoV vaccine is desirable to limit the impact of spillover of SARS-CoV-2-like sarbecoviruses. Multiple approaches are being investigated to induce a broad anti-sarbecovirus response, including mosaic RBD nanoparticles and mRNA vaccines.^25^^,^^26^ To induce a broad response, antibodies will need to target conserved regions of the S protein. While protein conservation across sarbecoviruses can be predicted, glycan processing cannot. We selected sarbecoviruses that present a high risk of human spillover to investigate the extent of glycan position and processing conservation. To this end, the genes for sarbecovirus S proteins were modified to introduce proline substitutions that have been successfully employed previously to generate soluble native-like trimers of S glycoproteins, some of which are used in existing SARS-CoV-2 immunogens.^27^^,^^28^^,^^29^ The resultant soluble S glycoproteins were purified, and glycosylation was analyzed by liquid chromatography-mass spectrometry (LC-MS). The N-linked glycan sites located in the NTD varied in position and in the abundance of oligomannose-type glycans. In contrast, an abundance of complex-type glycans on the S2 subunit was observed across all sarbecoviruses analyzed. To contextualize the changes in glycosylation, we generated structural models of the sarbecoviruses and modeled representative glycans onto the structure to map the 3D environment surrounding the N-linked glycan sites. This analysis revealed that the majority of divergent glycosylation patterns occurred on or proximal to the RBD, such as at N165, suggesting that subtle changes in the amino acid sequence in these regions can have cascading impacts on glycosylation of the S protein. These data support observations that the antibodies targeting the S2 region of the protein have the potential to provide a breadth of protection against a range of sarbecoviruses.

## Results

### Comparison of the N-linked glycan positions on sarbecoviruses

**Figure 1:**
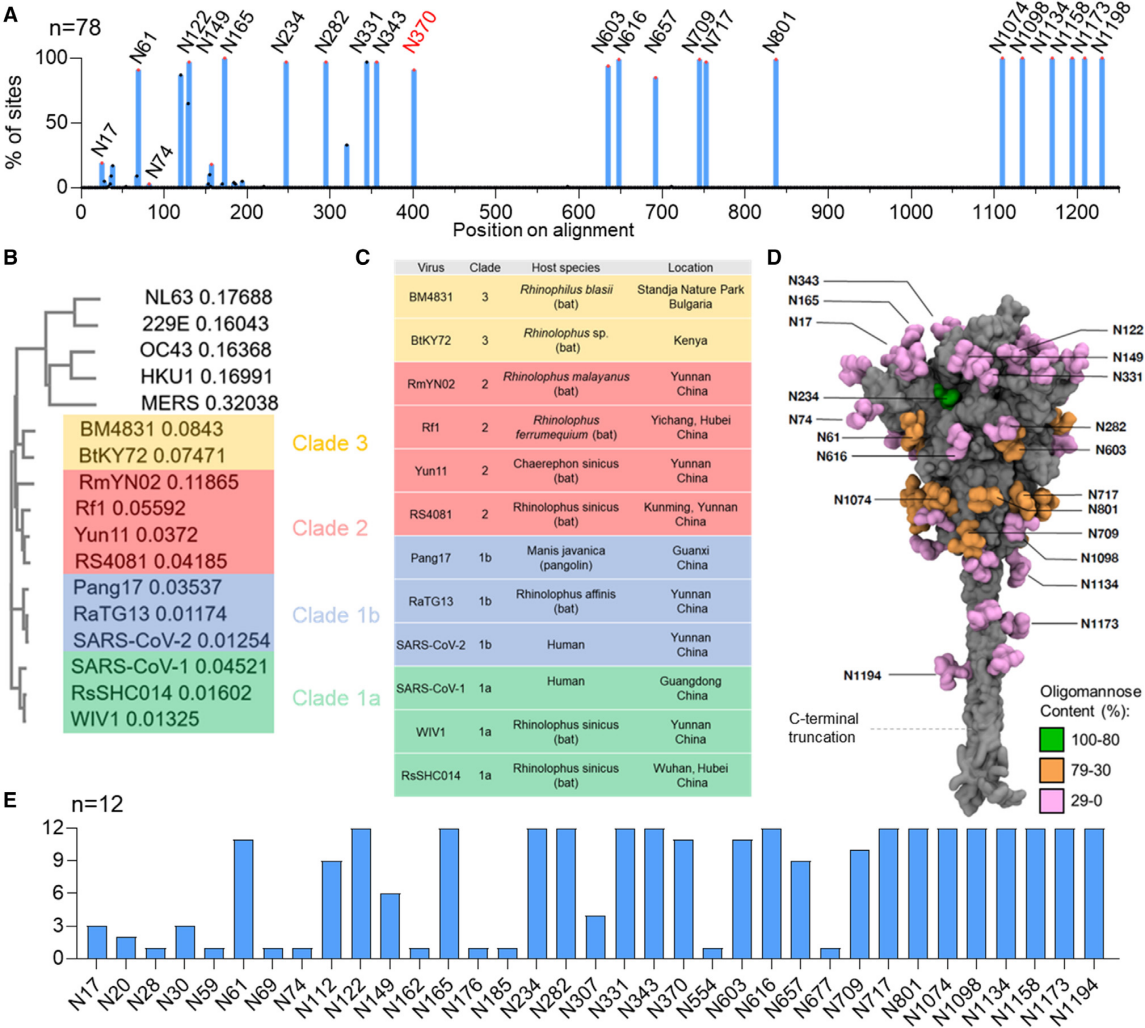
Conservation of N-linked glycosylation sequons across a sample of sarbecovirus S proteins (A) Alignment of 78 sarbecovirus S protein sequences. The y axis represents the proportion of sarbecoviruses that possess an N-linked glycan attachment site, expressed as a percentage of the total sequences used. Peaks corresponding to glycan sites from SARS-CoV-2 are labeled in black with their position on SARS-CoV-2. N370 is colored red because it is highly conserved but not present in SARS-CoV-2. (B) Clustal Omega multiple sequence alignment of the sarbecoviruses analyzed in this study alongside other human CoVs. Each sarbecovirus is colored according to the clade, which has been classified previously.^26^^,^^30^ (C) Table of the sarbecoviruses analyzed in this study, displaying the name, the species from which it was isolated, and the region in which the isolate was discovered. (D) Reproduction of a model of the SARS-CoV-2 glycan shield from Allen et al.^31^ determined from aggregation of data from recombinant proteins from multiple sources. The protein is displayed in gray, and the glycans are colored according to the abundance of oligomannose-type glycans present at each site. (E) Bar chart depicting the number of sarbecoviruses containing an NxS/T motif within the subpanel selected for glycopeptide analysis. Each sarbecovirus was aligned to SARS-CoV-2, and the glycan sites are displayed relative to their position on SARS-CoV-2. See also Tables S1–S3.

To compare the presence and location of potential N-linked glycosylation sites (PNGSs) across sarbecovirus S proteins, protein sequences for the S protein of 78 sarbecoviruses were obtained from the UniProt database. All S protein sequences were aligned using Clustal Omega. The sequence alignment and list of sarbecovirus S proteins used in this study can be found in Data S1. The sarbecoviruses used in this panel had protein sequence identities ranging from 55%–100% and included bat and pangolin sarbecoviruses. All sequences were aligned and searched for N-linked glycosylation sequons. To facilitate comparison with SARS-CoV-2 glycosylation, the sequences of the sarbecovirus S proteins were aligned with that of SARS-CoV-2, and throughout the manuscript, individual sites will be referred to based on their aligned position relative to SARS-CoV-2 (Figures 1A and 1D; Table S1; Data S1). When a sequon overlapped with SARS-CoV-2 S, it was included in Table S1. In this manner, the conservation of N-linked glycan sites could be compared across the 78 sarbecoviruses (Figure 1A).

The NTD displayed the most variation with regards to potential N-linked glycosylation (PNGS) conservation. This can be seen in the low conservation of the NTD SARS-CoV-2 PNGS N17 (19%), N74 (3%), and N146 (18%) (Table S1). Additional poorly conserved sites are located in the NTD of other sarbecoviruses, further demonstrating the variability in PNGS location in this region (Figure 1A). Outside of the NTD, almost all glycan sites are conserved in all sarbecoviruses used in this study, with only N657 displaying a conservation of less than 90%. Key regions of conservation include the N234 site, which was found in 97% of strains analyzed (Data S1), and the two glycan sites located on the SARS-CoV-2 RBD, N331 and N343, which were found in 97% of the 78-sample panel. Additionally, the N-linked glycan sites on the S2 portion of the protein were conserved on all strains analyzed. An additional point of note is the high conservation of N370, which is not present in SARS-CoV-2 but was located in 91% of sarbecoviruses analyzed in this study. This is notable because lack of this glycan has been shown to enhance SARS-CoV-2 infectivity relative to other sarbecoviruses.^32^^,^^33^ This 78-virus panel demonstrates that PNGS location is most variable in the NTD. However, across the rest of the protein, PNGS location is broadly conserved, with more than 90% of strains analyzed containing PNGSs located in the SARS-CoV-2 S2 domain.

The sarbecoviruses used in the 78-virus panel represent a diverse range of viruses with divergent receptor usage and hosts. In this study, we wanted to focus on sarbecoviruses that pose a threat regarding human spillover. This includes the ability to replicate within human cells, the ability to bind to the human ACE2 receptor, and an overall high sequence similarity to SARS-CoV-2. We therefore selected SARS-CoV-1, WIV1, and RsSHC014 (clade 1a); pang17, RaTG13, and SARS-CoV-2 (clade 1b); RmYN02, Rf1, Yun11, and RS4081 (clade 2); and BM4831 and BtKY72 (clade 3) for further study (Figures 1B and 1C)^20^^,^^22^^,^^23^^,^^34^ The selected isolates varied in sequence similarity from 70%–98% compared with SARS-CoV-2 at the amino acid level of the S glycoprotein (Table S2), demonstrating an overall high sequence conservation with SARS-CoV-2 compared with endemic CoVs circulating in humans, such as OC43, with a 35% sequence conservation relative to SARS-CoV-2. The smaller panel of sarbecoviruses chosen for further study recapitulated the glycosylation positions in the larger sarbecovirus panel displayed in Figure 1A. In both sets of sequences, the only glycan sequons that were present in less than 90% of sequences were N17, N74, N149, and N657. The only discrepancy was N709, which was present in 99% of the sequences in the 78-virus panel and 83% in the 12-virus panel (Table S3). This suggests that the 12 viruses selected for glycomics analyses will be representative of a larger subset of sarbecoviruses circulating in nature.

### Determination of the glycan processing state of sarbecovirus glycan sites

To investigate the variability of the sarbecovirus glycan shield, we selected 11 sarbecovirus S glycoprotein genes and introduced mutations to produce stabilized soluble trimers, using double proline substitutions (2P), a GSAS linker, and a C-terminal trimerization motif. Plasmids encoding the S glycoproteins were transfected into human embryonic kidney (HEK) 293F cells, and the soluble S glycoproteins were purified from the supernatant using nickel affinity chromatography followed by size exclusion chromatography (SEC). The size exclusion chromatogram displayed a single peak, representing S glycoprotein trimers.

**Figure 2:**
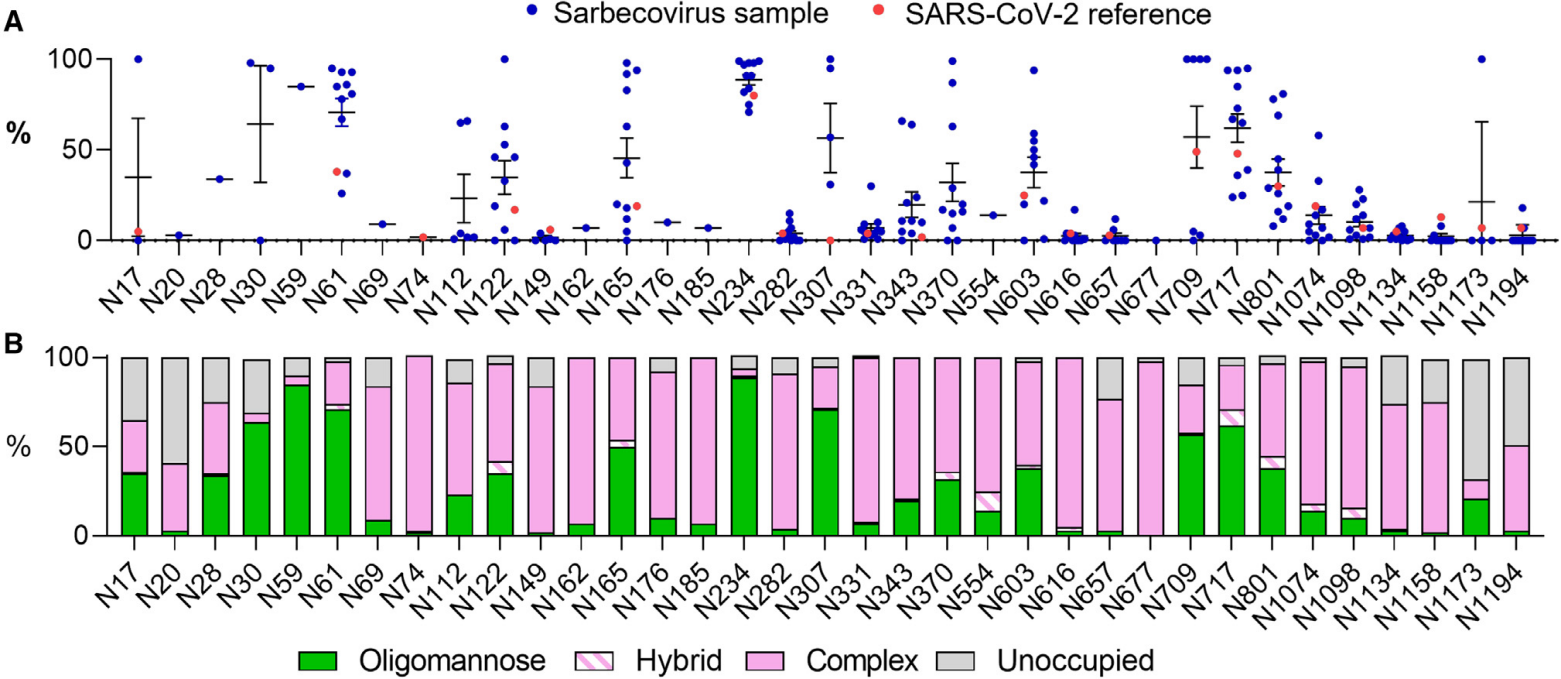
Determination of site-specific glycosylation of sarbecoviruses by LC-MS (A) Sum of the oligomannose-type glycans located at each N-linked glycan site on the sarbecoviruses analyzed in this study. The sequences for all sarbecoviruses were aligned with the SARS-CoV-2 S protein, and the glycan sites are presented aligned to this protein. The oligomannose-type glycan content of previously published site-specific data for SARS-CoV-2 S protein is shown as red dots.^31^ The mean of all strains is displayed as a line, and the error bars represent ±SEM or, when only two datasets are present, the range of the two datasets. (B) The averaged glycan processing state of all sarbecoviruses aligned with the SARS-CoV-2 S protein. Glycans classified as oligomannose-type are colored green, and hybrid-type glycans are represented as a white bar with pink hatches. Complex-type glycans are colored pink and the proportion of unoccupied N-linked glycan sites is displayed in gray. See also Figure S1 and Tables S4–S7.

We investigated the highlighted sarbecoviruses in Figure 1; however, the analysis of SARS-CoV-2 S protein was obtained from a previous publication.^31^ Three aliquots of the S glycoproteins were treated separately with trypsin, chymotrypsin, and alpha-lytic protease with the goal of generating glycopeptides containing a single N-linked glycan site. This enables the glycan processing state of each site to be investigated in a site-specific manner. Following analysis by LC-MS, the compositions of N-linked glycans were determined and then categorized based on the detected compositions to facilitate comparisons between the different samples. Full glycopeptide identification for each sample can be found in Data S2. Compositions corresponding to oligomannose-type glycans are distinct from others because they contain only two N-acetylglucosamine (GlcNAc) residues, whereas complex-type glycans contain at least three. Hybrid-type glycans were defined by the presence of 3–4 HexNAc residues and 5–6 hexose residues, distinguishing them from complex-type glycans. In this way, we identified the proportion of oligomannose-type glycans at each site for each sarbecovirus (Figure 2A). This analysis revealed that, although the positions of N-linked glycosylation sites are conserved between sarbecoviruses, the glycan processing of these sites can be highly variable. In addition, there are key sites that display remarkable conservation across all samples analyzed.

The N234 site is located within a sterically restricted environment, proximal to the RBD at the protomer interface. This glycan has been shown to have important roles in stabilizing the protein fold, controlling RBD dynamics, and removal of this glycan site diminishes the affinity to ACE2.^35^^,^^36^ In all sarbecoviruses analyzed, N234 was occupied by oligomannose-type glycans, ranging from 71% for RS4081 to 99% for BtKY72 (Figure 2A; Tables S6 and S7). The conservation of glycan processing provides further evidence of the key role of this glycan in the structure and function of not only SARS-CoV-2 but a broad range of sarbecoviruses. Similarly, the N282 glycan is conserved among all sarbecoviruses analyzed but is almost fully occupied by complex-type glycans. The role of this glycan in the structure and function of the S protein is less explored, but the conservation of this site could have important implications because of its proximity to the RBD.

In addition to the conserved oligomannose-type glycans located at N234, another remarkable region of conservation is the C terminus of the S protein in the S2 domain. The S2 domain spans N1074–N1194 and is the portion of the S protein that follows the furin cleavage site. While N1074 displays variable processing between sarbecoviruses (Figure 2A), sites N1098, N1134, N1158, N1173, and N1194 display low levels of oligomannose-type glycans, with each site containing under 33% oligomannose-type glycans on all samples analyzed, with one outlier at N1173. The processing of the complex-type glycans was more extensive than on other regions of the glycoprotein; for example, the most abundant glycan category detected at N1194 consisted of 6 N-acetyl hexosamines (Figure S1). This composition likely corresponds to large multi-antennary glycans and represents extensive glycan processing. The low abundance of oligomannose-type glycans in this region, combined with the conservation of N1074–N1194 in all sarbecoviruses analyzed, suggests that this region of the glycan shield is not only sparse but also conserved. Previous studies have highlighted that a lower oligomannose-type glycan content on viral glycoproteins correlates with a less dense glycan shield, which, in turn, will likely expose the underlying protein to antibodies.^37^ Therefore, the C-terminal portion of the S2 region likely presents conserved and exposed epitopes to the humoral immune system.

Despite regions of conservation in glycan processing, there were other conserved glycosylation sites, such as N801, that displayed variable glycan processing. To contextualize the observed differences in the site-specific glycosylation data, we calculated the “consensus” glycosylation across all samples, aligned with the SARS-CoV-2 N-linked glycosylation sites (Figures 2B and S1). Presenting the data in this way enables general trends in glycan processing to be discussed, which can then be compared with outliers within specific strains. This analysis revealed that the glycan processing state of sarbecoviruses is heterogeneous, with oligomannose-type glycans distributed across the S glycoprotein. Across all samples analyzed, the predominant glycoform detected was Man_5_GlcNAc_2_ (Figures 2B and S1). This is consistent with previous analyses of the SARS-CoV-2 S glycoprotein.^7^^,^^38^^,^^39^ This glycan is an intermediate processing state and is typically present in the *cis*-Golgi apparatus. On the majority of host glycoproteins, this glycan is further processed by the activity of GlcNAc transferase I (GNTI), which then enables assembly of complex and hybrid-type glycans. This glycan processing bottleneck suggests that the activity of this enzyme is sensitive to the steric environment surrounding the glycan sites, more so than that of the endoplasmic reticulum (ER)- and Golgi apparatus-resident mannosidases. It has been demonstrated previously that the activity of ER-α mannosidase I, which converts Man_9_GlcNAc_2_ to Man_8_GlcNAc_2_ can be sterically blocked by proximal glycans and protein and results in a high abundance of Man_9_GlcNAc_2_ on HIV-1 Env.^40^ The glycan shield of sarbecoviruses is less dense than that of HIV-1 Env,^37^ which likely means that ER-α mannosidase I is not inhibited to the same extent but that glycan density and protein steric effects are nevertheless sufficient to impede GnTI. This is a key observation for antibody binding because oligomannose-type glycan recognition by antibodies has been shown to favor alpha 1,2 mannose linkages,^41^^,^^42^ which are not present on Man_5_GlcNAc_2_. Additionally, complex-type glycans are found across the protein, with high levels of glycan processing occurring on the RBD glycan sites, N331 and N343. The low levels of oligomannose-type glycans around the RBD suggest that the glycan shield is sparse and that this domain is relatively flexible, as reflected by the lack of steric constraints placed on glycan processing of N331 and N343.

Interestingly, populations of N-linked glycan sites were detected that lacked glycan attachment toward the N and C terminus of the S proteins (Figure 2B). This phenomenon has been reported previously^31^^,^^43^^,^^44^ and likely occurs on the C terminus as a result of detachment of the translational machinery following translation termination. The processing of complex-type glycosylation with regard to elaboration with additional monosaccharides, such as sialic acid, is driven more by the producer cell used because the glycosyltransferase expression levels vary from cell to cell. Therefore, the complex-type glycan processing present on the sarbecovirus samples is reminiscent of viral glycoproteins analyzed previously from HEK293F cells (Data S2).^31^ Therefore, there are limits to the information that can be ascertained from analysis of complex-type glycans when derived from a recombinant source, requiring analysis of virus produced from appropriate cells of origin.

While regions of the glycan shield are highly conserved among the majority of sarbecoviruses analyzed, there are key glycan sites that are highly variable with respect to their glycan processing state, notably N61, N122, N165, N370, N717, and N801. Additional variability was observed at sites such as N17, N30, and N307; however, these sites were less conserved across the sarbecoviruses analyzed. The N165 site displays stark differences in the presentation of oligomannose-type glycans across different sarbecoviruses (Tables S4–S7). For example, Pang17, contains 98% oligomannose-type glycans at N165, whereas RaTG13 contains 5% at the same site (Figure 2A). The N165 glycan has been shown to have an important role in mediating the conformation of the RBD, facilitating the RBD-up position, which is favorable for receptor binding and also exposes neutralizing antibody epitopes. Therefore, changes in the processing of this glycan may be indicative of differential RBD dynamics between sarbecoviruses.^35^^,^^45^

### The extent of clade-specific glycan processing of sarbecoviruses

**Figure 3:**
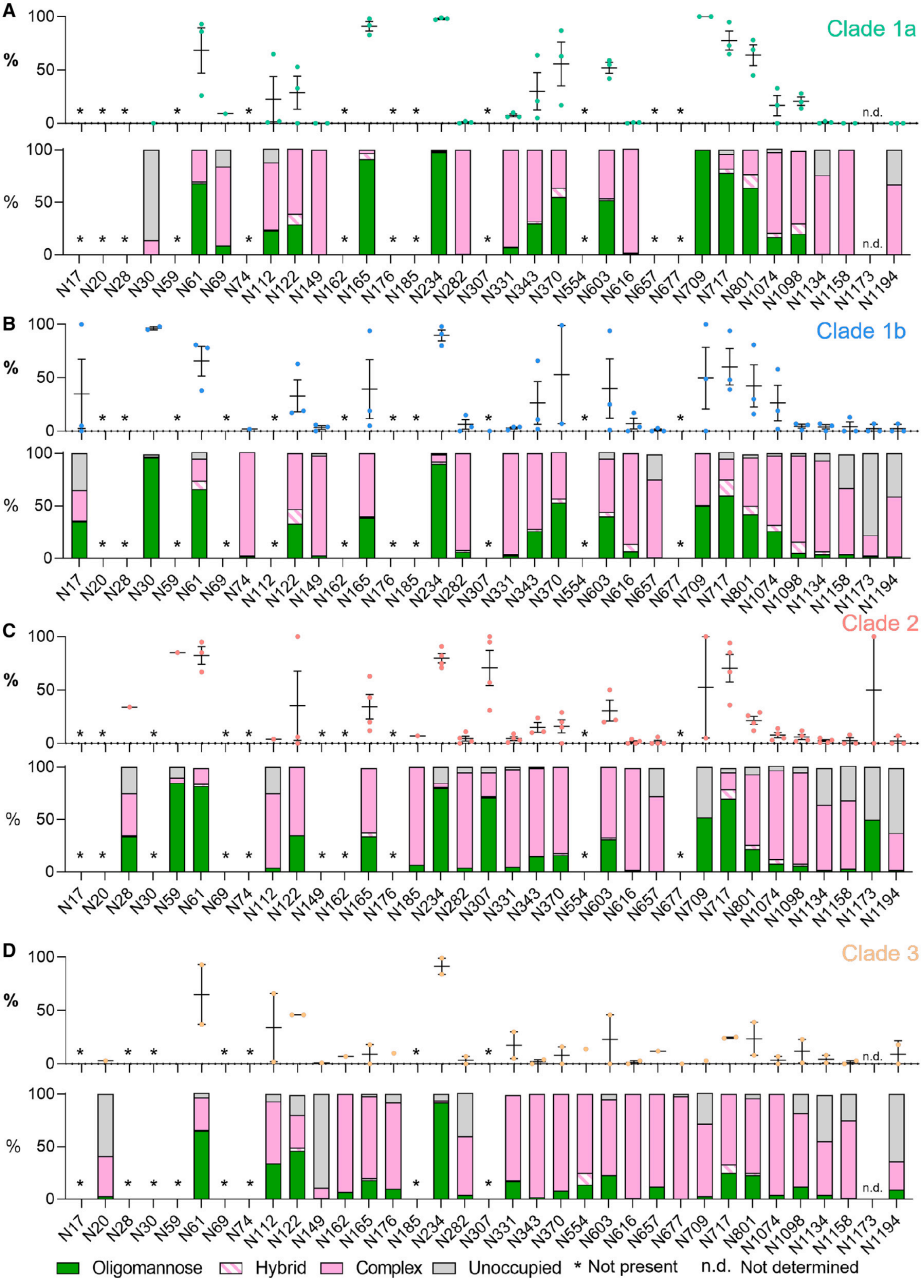
Clade-specific glycan processing of sarbecoviruses (A) Site-specific glycosylation of clade 1a sarbecoviruses, with the data displayed in a manner identical to Figure 2, with the symbols representing the oligomannose-type glycan content of individual strains and the bar graph representing the consensus glycosylation pattern at each site. (B) Site-specific glycosylation of clade 1b sarbecoviruses. (C) Site-specific glycosylation of clade 2 sarbecoviruses. (D) Site-specific glycosylation of clade 3 sarbecoviruses. Sites that are not present in a particular clade are labeled with an asterisk. Sites where the site-specific glycosylation could not be determined are labeled n.d. Error bars represent ± SEM. See also Tables S2–S7.

Because there are regions of the glycan shield of sarbecoviruses that are extremely variable, we sought to investigate whether sarbecoviruses from the same clade possess convergent glycan processing patterns. Using the classification outlined in Figure 1 we compared the site-specific glycosylation between sarbecoviruses in clade 1a, clade 1b, clade 2, and clade 3 (Figure 3). Clades 1a and 1b contained the highest proportion of oligomannose-type glycans (Figures 3A and 3B) and clade 3 the lowest (Figure 3D). This can be seen most prominently on glycan sites located toward the C terminus of the S1 domain, such as N717 and N801. In clade 1a, these sites are almost fully occupied by oligomannose-type glycans; for example, on clade 1a RsSCHC014, the N717 site contains 95% oligomannose-type glycans, whereas on BtkY72 of clade 3, the same site is only occupied by oligomannose-type glycans on 26% of sites (Tables S4 and S7). Sites such as N717 and N801 have been shown to form epitopes of glycan-binding antibodies that target oligomannose-type glycans.^41^ These data suggests that these glycan epitopes may not be conserved across sarbecoviruses and may not provide broad protection, although the antibodies may still bind at other regions of the trimer. An additional region of variable glycan processing is around the RBD. Of these sites, N165 is the most variable, with distinct glycan processing between clades. The clade 3 sarbecoviruses displayed the most processed N-linked glycans at N165 (18% oligomannose-type glycans) and clade 1a the least processed (91% oligomannose-type glycans). The processing at N165 in clade 1b and clade 2 is more variable, as demonstrated by the broad error bars in Figure 3. These data indicate that, despite broad conservation in the position of N-linked glycan sites, glycan processing can vary. This suggests that glycan position alone is not a predictor of glycan processing state. It is therefore important to understand the presentation of the glycan in its 3D environment to understand how the glycan shield can vary between different strains that are broadly conserved at the amino acid level.

### Mapping sarbecovirus glycan shields

**Figure 4:**
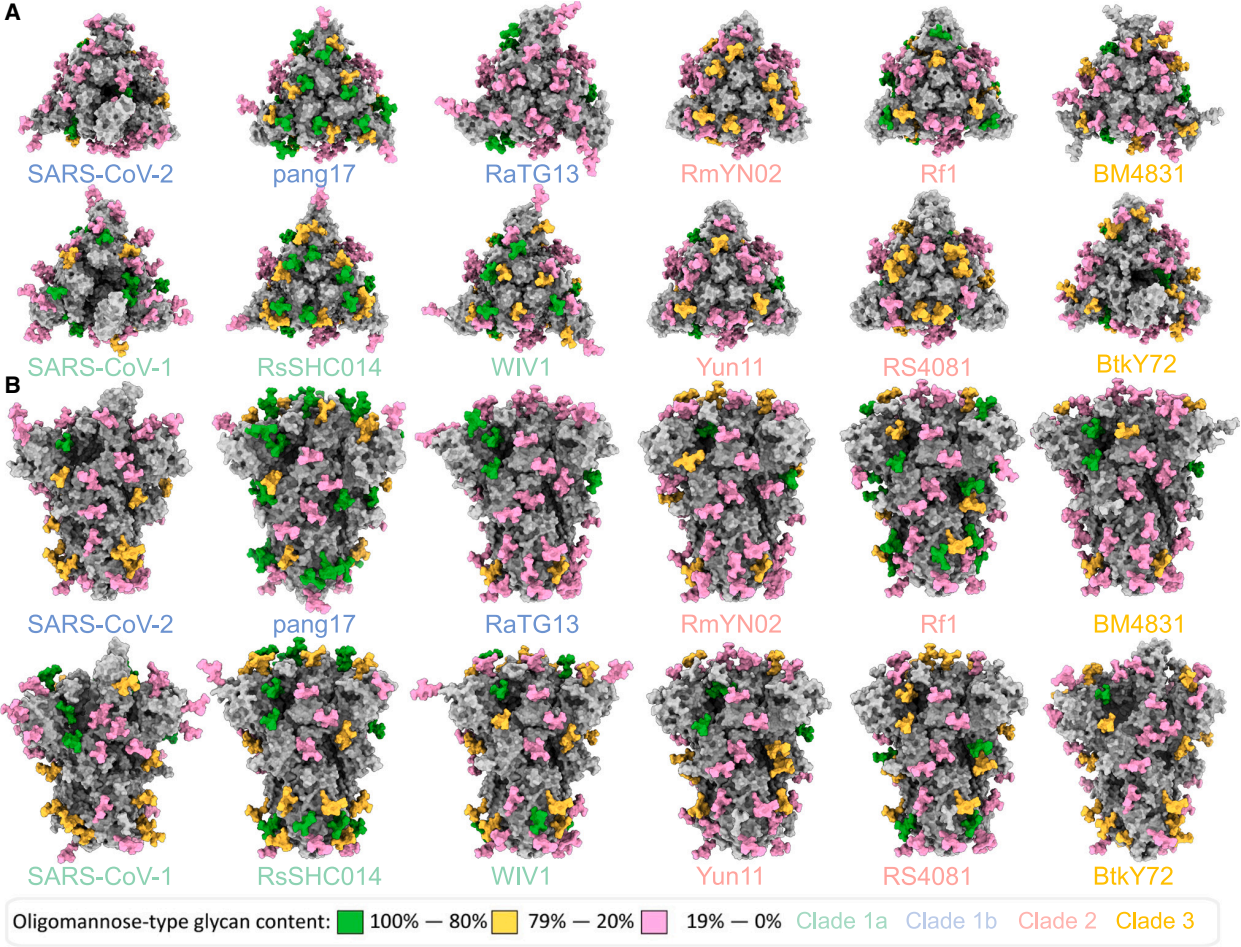
Modeling the glycan shield of sarbecoviruses with their site-specific glycosylation (A and B) 3D maps of the sarbecoviruses glycan shields are displayed top down (A) and side on (B). All models were constructed using SWISS-MODEL, GlycoShield, and the MS data displayed in Figure 3. Each model displays the protein sequence in gray. A representative Man_5_GlcNAc_2_ glycan was mapped onto each PNGS and is colored according to the oligomannose-type glycan content at each site, with 80% and above colored green, between 79% and 20% colored orange, and below 20% colored pink. The C-terminal region of the S protein was not resolved in the templates used to generate the models and therefore is not included.

Because the glycan shield varies in composition despite broad conservation of N-linked glycan sequon position, we sought to contextualize the site-specific glycosylation mapping the glycan shield onto the underlying protein structure (Figure 4).^46^^,^^47^^,^^48^^,^^49^The SWISS-MODEL template library (SMTL; v.2022-04-27, PDB release 2022-04-22) was searched with BLAST^50^ and HHblits^51^ for evolutionarily related structures matching the target sequence.

These templates do not contain glycans, and so we used an additional tool to attach a representative N-linked glycan at each site. Because Man_5_GlcNAc_2_ is the most abundant single composition on all samples, we modeled this glycan on every site using GlycoSHIELD.^52^ Any clashes were remodeled manually. This approach enabled 3D maps of the glycan shield to be generated for the 11 sarbecoviruses analyzed in this manuscript as well as for SARS-CoV-2, analyzed previously.^31^ Because the templates used to generate these maps did not contain a portion of the C-terminal domain, this was not included in our models, and the three C-terminal glycan sites are not included. Because N1158, N1773, and N1198 consist of almost exclusively complex-type glycans (Figure 2B), the processing of these sites is likely not influenced by glycan or protein clashes. Qualitatively, these models demonstrate the variability in the glycan shield that was shown with the site-specific analysis. Within clades, the glycan processing is variable. An example of this can be seen on clade 1b pang17 and the clade 1a sarbecovirus RsSHc014. Both sarbecovirus S proteins possess a higher proportion of oligomannose-type glycans at the trimer apex (Figure 4A) compared with the two other sarbecoviruses analyzed from these clades. RsSHC014 and pang17 contain elevated oligomannose-type glycans at N343, with ∼60% of the glycans at this site comprising oligomannose-type glycans on both S proteins (Tables S4 and S5). This suggests that, despite a high overall sequence similarity at the amino acid level, glycan processing is variable at and around the RBD. It is important to note that these models were generated based on previously resolved templates and do not represent experimentally determined structures. Fully glycosylated models of the sarbecoviruses can be found at https://doi.org/10.5281/zenodo.7636233.

### Variable glycan processing despite conservation of N-linked glycan site positions

**Figure 5:**
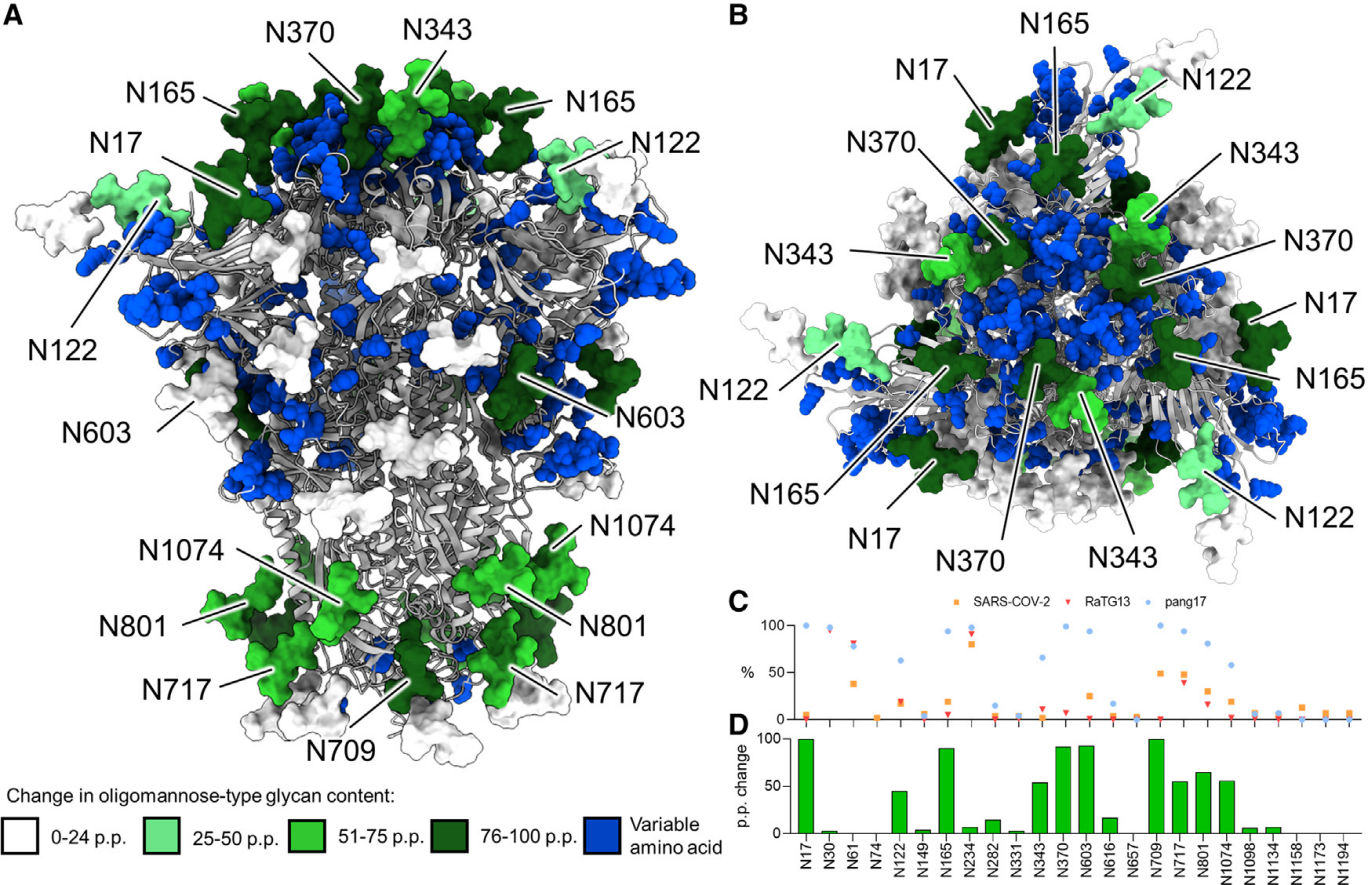
Glycan shield map of RaTG13-CoV to investigate the distinct glycan processing observed in clade 1b sarbecoviruses (A) Reproduction of the RaTG13 model generated in Figure 4, with the glycans recolored according to the p.p. difference in oligomannose-type glycans between RaTG13 and pang17, with a positive number representing a higher abundance of oligomannose-type glycans on pang17 relative to RaTG13. The protein sequence is displayed as a cartoon, with discrepancies in the amino acid sequence between RaTG13 and pang17 represented as blue spheres. Sites displaying increased oligomannose-type glycans on pang17 are labeled, with (A) representing a side-on view and (B) a top-down view. (C) Comparing the site-specific oligomannose-type glycan content of clade 1b sarbecoviruses: SARS-CoV-2, pang17, and RaTG13. (D) P.p. change in oligomannose-type glycans between RaTG13 and pang17. A positive p.p. change represents a glycoform that was present in higher abundance on pang17 compared with RaTG13.

From the 3D glycosylation maps generated, the starkest differences in glycosylation were observed in clade 1b. This clade includes SARS-CoV-2, RaTG13, and pang17, and therefore RaTG13 and pang17 have the highest sequence similarity in the S protein to SARS-Cov-2 (98% and 93%, respectively). As seen in Figure 3, the glycan processing of the clade 1b sarbecovirus S proteins used in this study was variable, most notably at N165, N343, and N370. To investigate how such variation in glycosylation can occur with only a small deviation in sequence identity, we directly compared the site-specific glycosylation of clade 1b sarbecoviruses (Figure 5). With regard to the glycan processing of conserved glycan sequons, the glycosylation of SARS-CoV-2 and RaTG13 was analogous; for example, N165 contained less than 20% oligomannose-type glycans in both samples (Figure 5B). In contrast, the pang17 S protein diverged at several regions across the glycan shield. RaTG13 and pang17 have an identical number and position of N-linked glycan sequons, whereas SARS-CoV-2 lacks the N30 and N370 sites but contains N74. Because pang17 and RaTG13 have the same number and position of glycosylation sites, the divergent glycan processing must result from other features of the protein.

To understand how the observed variability in glycan processing could be arising, we utilized the 3D glycosylation maps generated in Figure 4. In addition to the N-linked glycosylation sites, we compared regions of the protein sequence that differed between RaTG13 and pang17 (Figure 5A). To compare the site-specific presentation of oligomannose-type glycans, we determined the percentage point difference in glycosylation between pang17 and RaTG13. This represents the arithmetic difference in percentage values (pang17 – RaTG13), with a positive value representing a glycoform that is found in greater abundance on pang17. Across the pang17 S glycoprotein, there was an average 35 percentage point (p.p.) higher abundance of oligomannose-type glycans compared with RaTG13. This includes N122 (45 p.p.), N165 (90 p.p.), N343 (55 p.p.), N370 (90 p.p.), N603 (93 p.p.), N709 (100 p.p.), N717 (55 p.p.), N801 (65 p.p.), and N1074 (56 p.p.) (Figure 5B). Other regions of the glycan shield are conserved, such as the presentation of oligomannose-type glycans at N234 and more processed regions at N282 and the C-terminal sites.

Highlighted in blue in Figure 5 are amino acids that differ between RaTG13 and pang17. While the majority of amino acids are conserved with 93.19% sequence identity (Table S2), there are clusters of variable amino acids across the S. Variable amino acids cluster around the RBD domain in a similar manner as the accumulation of mutations on the emergent SARS-CoV-2 variants. These amino acid substitutions are located close to sites that display an elevation of oligomannose-type glycans on pang17 (Figure 5A, top panel). This includes N165, N370, and N343, which are located in and around the RBD, with an average increase of 79 p.p. Additionally, the N603 glycan site displays a similar increase in oligomannose-type glycans, and the amino acids around this region are variable as well. There are several sites toward the C terminus, including N709, N717, and N801, that show an elevation in oligomannose-type glycans; however, the protein sequence in this region is conserved. The increase in oligomannose-type glycans at these sites is not as pronounced as around the apex, and RaTG13 and SARS-CoV-2 contain oligomannose-type glycans at these sites. These results demonstrate that, despite broad conservation of amino acids across clade 1b sarbecoviruses, a limited number of mutations in key regions of the S are impacting the glycan shield. Because changing levels of oligomannose-type glycans can act as reporters for changes in the protein architecture,^31^^,^^53^ these results suggest that changes in the amino acid structures that modulate the structure of the protein will have impacts on glycan processing across the glycan shield. Sites such as N165 have been shown to be sensitive to changes in the protein architecture. For example, introduction of additional stabilizing mutations into the Wuhan hu1 SARS-CoV-2 S, termed HexaPro, caused an increase in oligomannose-type glycans at N165 in a manner comparable with that observed when comparing pang17 S protein with the SARS-CoV-2 S protein.^45^^,^^54^

## Discussion

The propensity of SARS-CoV-2 to mutate and generate new variants of concern highlights the importance of investigating the molecular architecture of similar sarbecoviruses to prepare for a potential species crossover in the future. The sarbecoviruses investigated in this study share similar sequences with SARS-CoV-2 and are being investigated for use in formats for pan-sarbecovirus vaccine candidates.^55^ The goal of this study was to investigate the variability in the glycan shield of sarbecoviruses because they constitute one-third of the mass of the surface of the S glycoprotein, and alterations in their presence and processing will likely alter the antigenic surface of the viral S. With regard to the position of PNGSs, the majority of sites were conserved with SARS-CoV-2. The N-terminal region displayed the most variability, with sites such as N17 and N74 not seen on many of the sarbecoviruses analyzed in this study. Analysis of the glycan processing of the sarbecoviruses revealed regions of conserved and divergent glycan processing. The N234 site likely plays a key role in the stability and function of the S protein,^35^ and its position and processing state were conserved across all samples analyzed. The most remarkable conservation was observed in the S2 region of the protein, with sites N1074, N1098, N1134, N1158, N1173, and N1198 conserved across all samples. These sites also possessed low levels of oligomannose-type glycans, suggesting that steric constraints on glycan-processing enzymes resulting from protein/glycan clashes are low. Conversely, some regions display highly divergent glycan processing, with the conserved N165 glycan site displaying extensive variability in the abundance of oligomannose-type glycans, suggesting variable protein architecture around this region of the protein. We generated 3D maps of the sarbecovirus glycan shields to contextualize the changes in glycosylation, and for three highly similar sarbecoviruses (SARS-CoV-2, RaTG13, and pang17), we showed that slight modifications in the amino acid sequence can result in distinct glycosylation profiles, most notably on and around the RBD.

Our observations provide insight into regions that may prove more promising in the design of pan-sarbecovirus vaccines, we demonstrate that the position and processing of glycans in and around the RBD in the NTD vary across sarbecoviruses, even amongst S proteins that have more than 90% conservation at the amino acid level, such as pang17 and SARS-CoV-2. This is also seen in the continued evolution of SARS-CoV-2, where mutations in the S protein are focused on the RBD and S1 domains. This is in response to these regions being the immunodominant regions of the S glycoprotein, and subtle changes in this region can diminish the ability of neutralizing antibodies to recognize new variants. Conversely, glycosylation in the S2 domain is conserved and is of the complex type. This suggests that this region of the protein is antigenically conserved and that the glycan shield density in this region is low. Therefore, the S2 domain may provide a more attractive target for vaccine design. Indeed, several studies have highlighted the potential for broad CoV antibody recognition and neutralization by exploiting this domain.^56^^,^^57^^,^^58^^,^^59^^,^^60^ It is important to note that these antibodies are not as potent as RBD-specific neutralizing antibodies. The discovery of many CoVs in animal reservoirs suggests that, in a manner similar to influenza, CoV-induced pandemics are of considerable likelihood in the future, and understanding the antigenic surface of these viruses and how it can change is important to consider when preparing for future outbreaks.

### Limitations of the study

This study focuses on features of glycosylation that are largely directed by the structural properties of the protein, particularly the levels of oligomannose-type glycans. While previous analyses comparing the glycosylation of recombinant SARS-CoV-2 S protein with virally derived SARS-CoV-2 S protein has shown consistent glycan processing,^31^^,^^39^ the use of recombinant systems will likely influence glycosylation of the S protein, particularly with respect to terminal processing of complex-type glycans. Biologically, there are likely to be substantial differences in glycosylation, particularly in the nature of complex-type glycans, depending on the cellular source of the virus and the local inflammatory environment. Our study investigates sarbecoviruses from a range of different animal hosts, and natural glycosylation will be species specific, undermining the ability to have a standardized experimental system for comparison. Here, the protein-specific effects are emphasized by adopting a standardized recombinant approach with a single cell line.

## STAR★Methods

### Key resources table

**Table:** 

REAGENT or RESOURCE	SOURCE	IDENTIFIER
**Biological samples**		

phCMV3 vector	Addgene	https://www.addgene.org/vector-database/6216/

**Chemicals, peptides, and recombinant proteins**		

Acetonitrile, 80%, 20% Water with 0.1% Formic Acid, Optima LC/MS	Fisher Scientific	Cat# 15431423
Water with 0.1% Formic Acid (v/v), Optima™ LC/MS Grade	Fisher Scientific	Cat# LS118-212
Acetonitrile	Fisher Scientific	Cat# 10489553
Trifluoroacetic acid	Fisher Scientific	Cat# 10155347
Dithiothreitol	Sigma-Aldrich	Cat# 43819
Iodacetamide	Sigma-Aldrich	Cat# I1149
Mass spectrometry grade trypsin	Promega	Cat# V5280
Sequencing grade chymotrypsin	Promega	Cat# V1061
Urea	Sigma-Aldrich	U5378-1KG
Transfectagro	Corning	Product Number40-300-CV
40K polyethylenimine (PEI)	Sigma Aldrich	CAS Number: 49553-93-7
HisPur Ni-NTA resin	Thermo Fisher Scientific	90092
Superdex 200	Cytiva	28990944

**Critical commercial assays**		

GeneArt	Thermo Fisher Scientific	https://www.thermofisher.com/uk/en/home/life-science/cloning/c-misc/geneart
Gibson assembly	NEB	https://international.neb.com/applications/cloning-and-synthetic-biology/dna-assembly-and-cloning/gibson-assembly

**Deposited data**		

Glycosylated SARS-CoV-2 model	Zuzic et al. 2021	https://doi.org/10.5281/zenodo.5760159
Mass spectrometry data	This paper	ftp://massive.ucsd.edu/MSV000090155/
Glycosylated sarbecovirus models	This paper	https://doi.org/10.5281/zenodo.7015311

**Experimental models: Cell lines**		

HEK 293F cells	Thermo Fisher Scientific	Cat# R79007

**Recombinant DNA**		

Yun11-His-Avi spike	Raiees Andrabi TSRI (this paper)	N/A
10B-BM4831-His-Avi spike	Raiees Andrabi TSRI (this paper)	N/A
10C-BtKY72-His-Avi spike	Raiees Andrabi TSRI (this paper)	N/A
10D-Pang17-His-Avi spike	Raiees Andrabi TSRI (this paper)	N/A
10E-RaTG13-His-Avi spike	Raiees Andrabi TSRI (this paper)	N/A
10F-Rf1-His-Avi spike	Raiees Andrabi TSRI (this paper)	N/A
10G-RmYN02-His-Avi spike	Raiees Andrabi TSRI (this paper)	N/A
10H-RS4081-His-Avi spike	Raiees Andrabi TSRI (this paper)	N/A
10I-RsSHC014-His-Avi spike	Raiees Andrabi TSRI (this paper)	N/A
10J-WIV1-His-Avi spike	Raiees Andrabi TSRI (this paper)	N/A
SARS-CoV-2-His-Avi	Raiees Andrabi TSRI (this paper)	N/A
SARS-CoV-1-His-Avi	Raiees Andrabi TSRI (this paper)	N/A

**Software and algorithms**		

Byos™ (Version 4.0)	Protein Metrics Inc.	https://www.proteinmetrics.com/products/byonic/
UCSF Chimera (version 1.4)	UCSF	https://www.cgl.ucsf.edu/chimera/download.html
Coot (version 0.9-pre)	MRC Laboratory of Molecular Biology	https://www2.mrc-lmb.cam.ac.uk/personal/pemsley/coot/
Pymol (version 2.5.0)	Schrödinger	https://pymol.org/2/
GlycoSHIELD	https://doi.org/10.1101/2021.08.04.455134	https://github.com/GlycoSHIELD-MD/GlycoSHIELD-0.1
XCalibur Version v4.2	Thermo Fisher	N/A
Orbitrap Fusion Tune application v3.1	Thermo Fisher	N/A
GraphPad Prism v8	GraphPad	N/A
Clustal Omega	Clustal	http://www.clustal.org/omega/

**Other**		

C18 ZipTip	Merck Milipore	Cat# ZTC18S008
Vivaspin 500, 3 kDa MWCO, Polyethersulfone	Sigma-Aldrich	Cat# GE28-9322-18
Orbitrap Eclipse mass spectrometer	Thermo Fisher Scientific	N/A
Ultimate 3000 HPLC	Thermo Fisher Scientific	N/A
EasySpray PepMap RSLC C18 column (75 μm × 75 cm)	Thermo Fisher Scientific	Cat# ES805
PepMap™ Neo Trap Cartridge	Thermo Fisher Scientific	Catalog number: 174500

### Resource availability

#### Lead contact

Any further information and requests should be directed to and will be fulfilled by the lead contact, Max Crispin (max.crispin@soton.ac.uk).

#### Materials availability

The spike constructs include SARS-CoV-2 (residues 1–1208; GenBank ID: MN908947), SARS-CoV-1 (residues 1–1190; GenBank ID: AAP13567), RaTG13 (residues 1-1204, GenBank ID: QHR63300.2), Pang17 (residues 1-1202, GenBank ID: QIA48632.1), WIV1 (residues 1-1191, GenBank ID: KF367457), RsSHC014 (residues 1-1191, GenBank ID: AGZ48806.1), BM48-31 (residues 1-1194, GenBank ID: NC_014470.1), BtKY72 (residues 1-1193, GenBank ID: KY352407), RmYN02 (residues 1-1165, GISAID ID: EPI_ISL_412977), Rf1 (residues 1-1176, GenBank ID: DQ412042.1), Rs4081 (residues 1-1176, GenBank ID: KY417143.1), Yun11 (residues 1-1176, GenBank ID: JX993988).

All reagents generated in this study are available from the Lead contact with a completed Materials Transfer Agreement, although the exact protein expression batch analyzed in this study has been used up and will require re-expression prior to sharing. Plasmids encoding for these proteins can be provided.

### Experimental model and subject details

#### DNA template design and protein production using HEK293F cells

To produce recombinant spike proteins, human embryonic kidney (HEK) 293F cells were used. The expression plasmids of soluble spike ectodomain proteins were constructed by DNA fragments synthesized at GeneArt (Thermo Fisher Scientific) followed by cloning into the phCMV3 vector by Gibson assembly. The soluble spike proteins were stabilized in the trimeric prefusion state by introducing double proline substitutions (2P) in the S2 subunit, replacing the furin cleavage sites by a GSAS linker, as well as incorporating the trimerization motif T4 fibritin at the C terminus of the spike proteins. The HRV-3C protease cleavage site, 6×His-Tag and AviTag spaced by GS linkers were added to the C terminus for protein purification and biotinylation.

For protein expression, 350ug of the plasmids encoding spikes were transfected into 1L HEK-293F cells at 1 million cells/ml using Transfectagro (Corning) and 40K polyethylenimine (PEI) (1 mg/mL). The plasmid and transfection reagents were combined and filtered before PEI was added. The mixture solution was incubated at room temperature for 20-30 min before being added into cells. After 4 days, the supernatant was centrifuged and filtered, followed by loading onto columns with HisPur Ni-NTA resin (Thermo Fisher Scientific). The resin-bound protein was washed (25 mM imidazole, pH 7.4) and eluted using 25 mL elution buffer (250 mM imidazole, pH 7.4). The eluate was buffer-exchanged into PBS and further purified through size-exclusion chromatography (SEC) by Superdex 200 (GE Healthcare).

### Method details

#### Potential N-linked glycan conservation and alignment search

To investigate the distribution of potential N-linked glycan sites on sarbecoviruses, the UniProt database was used to obtain sarbecovirus S protein sequences. A total of 78 sequences were obtained and were aligned using Clustal Omega. The aligned sequences were then searched for PNGS, and the percentage of sites was determined. The full list of sequences is available in Data S1.

#### Site-specific glycan analysis by LC-MS

Three aliquots of sarbecovirus were denatured for 1h in 50 mM Tris/HCl, pH 8.0 containing 6 M of urea and 5 mM dithiothreitol (DTT). The denatured proteins were alkylated by adding 20 mM iodoacetamide (IAA) and incubated for 1h in the dark, followed by a 1h incubation with 20 mM DTT to eliminate residual IAA. The alkylated Env proteins were buffer exchanged into 50 mM Tris/HCl, pH 8.0 using Vivaspin columns (3 kDa) and the aliquots were digested separately overnight using trypsin, chymotrypsin (Mass Spectrometry Grade, Promega) or alpha lytic protease (Sigma Aldrich) at a ratio of 1:30 (w/w). The next day, the peptides were dried and extracted using C18 Zip-tips (Merck Milipore). The peptides were dried again, re-suspended in 0.1% formic acid and analyzed by nanoLC-ESI MS with an Ultimate 3000 HPLC (Thermo Fisher Scientific) system coupled to an Orbitrap Eclipse mass spectrometer (Thermo Fisher Scientific) using stepped higher energy collision-induced dissociation (HCD) fragmentation. Peptides were separated using an EasySpray PepMap RSLC C18 column (75 μm × 75 cm). A trapping column (PepMap *Neo* Trap Cartridge) was used in line with the LC prior to separation with the analytical column. The LC conditions were as follows: 280-min linear gradient consisting of 4-32% acetonitrile in 0.1% formic acid over 260 min followed by 20 min of alternating 76% acetonitrile in 0.1% formic acid and 4% Acn in 0.1% formic acid, used to ensure all the sample had eluted from the column. The flow rate was set to 300 nL/min. The spray voltage was set to 2.5 kV and the temperature of the heated capillary was set to 55°C. The ion transfer tube temperature was set to 275°C. The scan range was 375–1500 m/z. Stepped HCD collision energy was set to 15, 25 and 45% and the MS2 for each energy was combined. Precursor and fragment detection were performed using an Orbitrap at a resolution MS1 = 120,000. MS2 = 30,000. The AGC target for MS1 was set to standard and injection time set to auto which involves the system setting the two parameters to maximize sensitivity while maintaining cycle time. Full LC and MS methodology can be extracted from the appropriate Raw file using XCalibur FreeStyle software or upon request.

Glycopeptide fragmentation data were extracted from the raw file using Byos (Version 3.5; Protein Metrics Inc.). The glycopeptide fragmentation data were evaluated manually for each glycopeptide; the peptide was scored as true-positive when the correct b and y fragment ions were observed along with oxonium ions corresponding to the glycan identified. The MS data was searched using the Protein Metrics 305 N-glycan library with sulfated glycans added manually. The relative amounts of each glycan at each site as well as the unoccupied proportion were determined by comparing the extracted chromatographic areas for different glycotypes with an identical peptide sequence. All charge states for a single glycopeptide were summed. The precursor mass tolerance was set at 4 ppm and 10 ppm for fragments. A 1% false discovery rate (FDR) was applied. The relative amounts of each glycan at each site as well as the unoccupied proportion were determined by comparing the extracted ion chromatographic areas for different glycopeptides with an identical peptide sequence. Glycans were categorized according to the composition detected.

HexNAc(2)Hex(10+) was defined as M9Glc, HexNAc(2)Hex(9−5) was classified as M9 to M3. Any of these structures containing a fucose were categorized as FM (fucosylated mannose). HexNAc(3)Hex(5−6)X was classified as Hybrid with HexNAc(3)Hex(5-6)Fuc(1)X classified as Fhybrid. Complex-type glycans were classified according to the number of HexNAc subunits and the presence or absence of fucosylation. As this fragmentation method does not provide linkage information compositional isomers are grouped, so for example a triantennary glycan contains HexNAc 5 but so does a biantennary glycans with a bisect. Core glycans refer to truncated structures smaller than M3. M9glc- M4 were classified as oligomannose-type glycans.

#### Model generation: Template search

Template search with BLAST and HHblits was performed against the SWISS-MODEL template library (SMTL, last update: 2022-04-27, last included PDB release: 2022-04-22). The target sequence was searched with BLAST against the primary amino acid sequence contained in the SMTL. An initial HHblits profile was built using the procedure outlined in,^51^ followed by 1 iteration of HHblits against Uniclust30.^61^ The obtained profile was then searched against all profiles of the SMTL.

#### Model generation: Model building

Models are built based on the target-template alignment using ProMod3.^49^ Coordinates which are conserved between the target and the template are copied from the template to the model. Insertions and deletions are remodeled using a fragment library. Side chains are then rebuilt. Finally, the geometry of the resulting model is regularized by using a force field. The global and per-residue model quality has been assessed using the QMEAN scoring function.^48^The quaternary structure annotation of the template is used to model the target sequence in its oligomeric form. The method^62^ is based on a supervised machine learning algorithm, Support Vector Machines (SVM), which combines interface conservation, structural clustering, and other template features to provide a quaternary structure quality estimate (QSQE). To map the N-linked glycans to the sarbecovirus templates GlycoSHIELD was used to graft glycan conformers derived from extensive molecular dynamics simulations.^52^ A representative N-linked glycan was used Man_5_GlcNac_2_. The grafting procedure was performed using a cutoff radius of 0.7 Å.

### Quantification and statistical analysis

Mass spectrometry data was analyzed using Byos™ (Version 4.0), including the identification of glycopeptides and the XIC quantification of different glycoforms with the same peptide sequence. All calculations and graphical representations of data were performed using GraphPad Prism v8. Molecular models of sarbecoviruses were visualized using UCSF Chimera (version 1.4).

## Data Availability

• Raw files, protein sequences and glycan library used for the site-specific glycan analysis are deposited on the MASSive server under the following ID: ftp://massive.ucsd.edu/MSV000090155/. Supplemental spreadsheets for the manuscript can be found on Mendeley Data https://doi.org/10.17632/4xjpmhj7xb.1. • Glycosylated models of the coronaviruses used in this study are deposited: https://doi.org/10.5281/zenodo.7636233 • This paper does not report original code. Any additional information required to reanalyze the data reported in this paper is available from the lead contact upon request.

## Supplemental information

Document S1. Figure S1 and Tables S1–S7

Data S1. Sarbecovirus sequence alignment

Data S2. Site-specific glycan analysis data

Document S2. Article plus supplemental information
