# Supplementary material for: The diversity of the glycan shield of sarbecoviruses related to SARS-CoV-2
Source: Cell Rep. 2023 Mar 15;42(4):112307. doi: 10.1016/j.celrep.2023.112307 (PMC10015101; doi:10.1016/j.celrep.2023.112307)
Supplement: Document S1. Figure S1 and Tables S1–S7 [file mmc1.pdf]

Cell Reports, Volume *42*

Supplemental information

The diversity of the glycan shield of sarbecoviruses related to SARS-CoV-2

Joel D. Allen, Dylan P. Ivory, Sophie Ge Song, Wan-ting He, Tazio Capozzola, Peter Yong, Dennis R. Burton, Raiees Andrabi, and Max Crispin

Supplemental Table 1: Conservation of SARS-CoV-2 PNGS across 78 sarbecovirus sequences (related to Figure 1)

Position on alignment	SARS-CoV-2 glycan position	% of sites
25	17	19%
69	61	91%
82	74	3%
130	122	97%
157	146	18%
173	165	100%
247	234	97%
295	282	97%
344	331	97%
356	343	97%
401	370	91%
635	603	94%
648	616	99%
692	657	85%
745	709	99%
753	717	97%
837	801	99%
1110	1074	100%
1134	1098	100%
1170	1134	100%
1194	1158	100%
1209	1173	100%
1230	1194	100%

Supplemental Table 2: Coronavirus sequence similarity displayed as a percentage identity matrix (related to Figure 1)

	NL63	229E	OC43	HKU1	MERS	BM4831	BtKY72	Pang17	RaTG13	SARS-CoV-2	RmYN02	SARS-CoV-1	RsSHC014	WIV1	Rf1	Yun11	RS4081
NL63	100	66	31	31	29	31	31	29	30	30	30	30	30	30	31	30	31
229E	66	100	32	33	32	31	32	31	32	32	32	32	32	32	32	32	32
OC43	31	32	100	67	37	36	35	35	35	35	36	35	36	36	37	36	36
HKU1	31	33	67	100	36	35	34	34	35	35	36	35	35	36	36	34	35
MERS	29	32	37	36	100	35	35	34	35	35	35	35	35	35	35	35	35
BM4831	31	31	36	35	35	100	84	73	73	73	72	76	76	76	75	76	76
BtKY72	31	32	35	34	35	84	100	74	74	74	73	77	77	77	77	78	77
Pang17	29	31	35	34	34	73	74	100	93	93	76	78	78	78	77	77	77
RaTG13	30	32	35	35	35	73	74	93	100	98	76	78	78	78	77	77	77
SARS2	30	32	35	35	35	73	74	93	98	100	76	77	78	78	77	77	77
RmYN02	30	32	36	36	35	72	73	76	76	76	100	74	74	74	79	79	79
SARS1	30	32	35	35	35	76	77	78	78	77	74	100	90	92	79	81	81
RsSHC014	30	32	36	35	35	76	77	78	78	78	74	90	100	97	80	81	81
WIV1	30	32	36	36	35	76	77	78	78	78	74	92	97	100	80	81	81
Rf1	31	32	37	36	35	75	77	77	77	77	79	79	80	80	100	90	89
Yun11	30	32	36	34	35	76	78	77	77	77	79	81	81	81	90	100	92
RS4081	31	32	36	35	35	76	77	77	77	77	79	81	81	81	89	92	100

Supplemental Table 3: Abundance of SARS-CoV-2 PNGS in the 78- and 12- virus panels (Related to Figure 1)

78-virus panel	% of sites
N165	100%
N1074	100%
N1098	100%
N1134	100%
N1158	100%
N1173	100%
N1194	100%
N616	99%
N709	99%
N801	99%
N122	97%
N234	97%
N282	97%
N331	97%
N343	97%
N717	97%
N603	94%
N61	91%
N370	91%
N657	85%
N17	19%
N149	18%
N74	3%

12-virus panel	Number of sites	
N122	12	100%
N165	12	100%
N234	12	100%
N282	12	100%
N331	12	100%
N343	12	100%
N616	12	100%
N717	12	100%
N801	12	100%
N1074	12	100%
N1098	12	100%
N1134	12	100%
N1158	12	100%
N1173	12	100%
N1194	12	100%
N61	11	92%
N370	11	92%
N603	11	92%
N709	10	83%
N657	9	75%
N149	6	50%
N17	3	25%
N74	1	8%

sites under 90 %

N657	85%
N17	19%
N149	18%
N74	3%

sites under 90 %

N709	10	83%
N657	9	75%
N149	6	50%
N17	3	25%
N74	1	8%

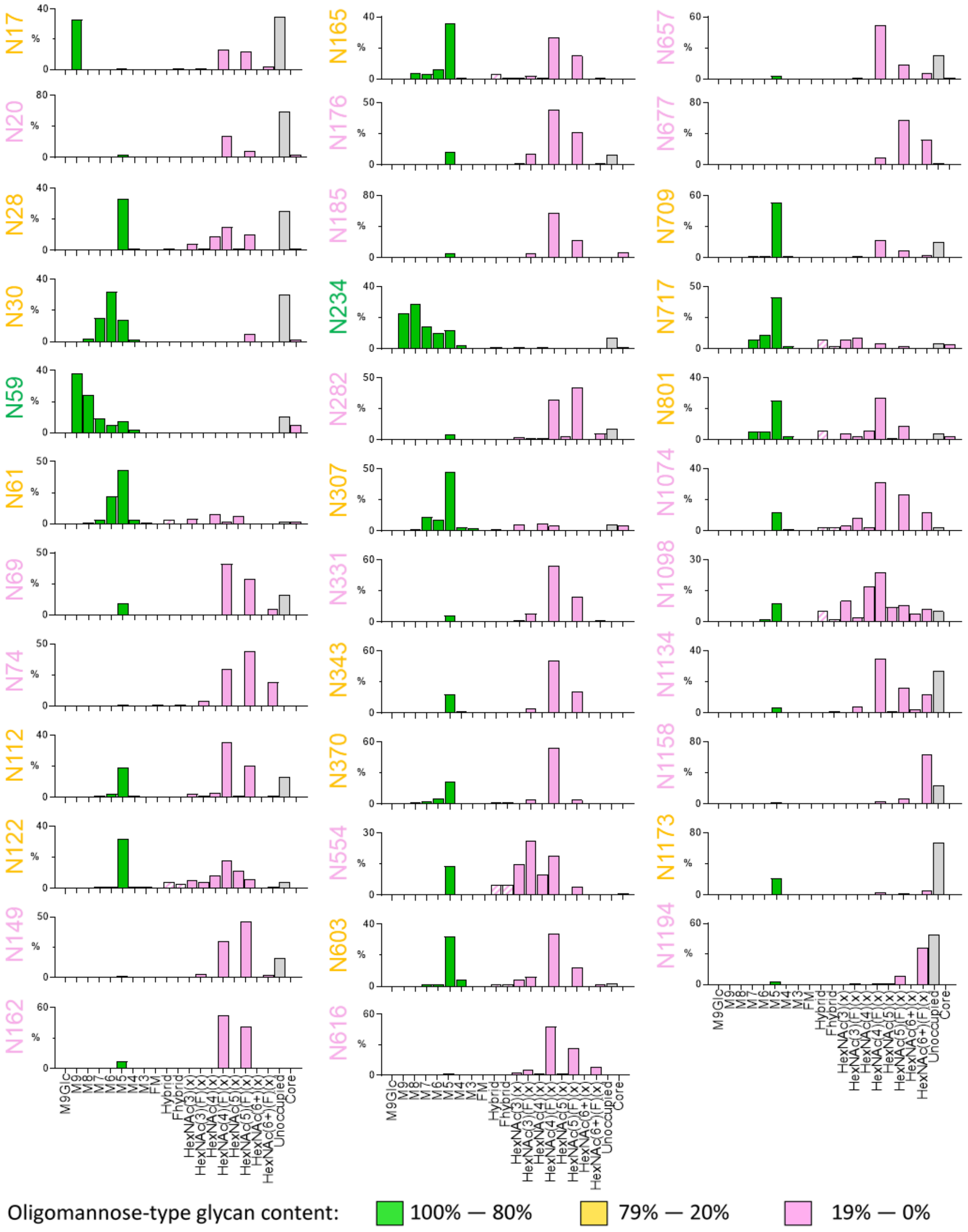

Supplemental Figure 1: Consensus site-specific glycan analysis of sarbecovirus glycan shields. The consensus site-specific glycosylation of all sarbecoviruses analyzed in this study. Each sarbecovirus was aligned to SARS-CoV-2 and where a conserved glycan site was present, the detected compositions were averaged. The compositions detected by LC-MS were grouped, as outlined in the materials and methods. Oligomannose-type glycans are displayed as green, hybrid-type glycans are displayed as white, with pink cross hatching and complex-type glycans displayed as pink bars. The proportion of unoccupied N-linked glycan sites is displayed as a grey bar. Each site label is colored according to the oligomannose-type glycan content. Related to Figure 2

Supplemental Table 4: Site-specific glycan analysis of clade 1a sarbecoviruses (related to Figure 2 and 3)

RsSHC014	N17	N20	N28	N30	N59	N61	N69	N74	N112	N122	N149	N162	N165	N176	N185	N234	N282	N307	N331	N343	N370	N554	N603	N616	N657	N677	N709	N717	N801	N1074	N1098	N1134	N1158	N1173	N1194
M9Glc						0			0	0	0		0			0	0		0	0	0		0	0			0	0	0	0	0	0	0	0	0
M9						0			0	0	0		4			77	0		0	0	2		0	0			0	0	0	0	0	0	0	0	0
M8						0			1	0	0		42			18	0		0	0	18		0	0			0	0	0	0	0	0	0	0	0
M7						0			3	4	0		25			2	0		0	0	13		0	0			0	21	15	0	0	0	0	0	0
M6						49			15	0	0		17			1	0		0	3	13		0	0			0	30	8	0	1	0	0	0	0
M5						37			41	48	0		9			0	2		5	52	41		52	1			98	41	44	32	19	1	0	0	0
M4						7			4	1	0		2			0	0		0	9	0		2	0			2	4	2	0	0	0	0	0	0
M3						2			0	0	0		0			0	0		0	1	0		1	0			0	0	1	0	0	0	0	0	0
FM						0			0	0	0		0			0	0		1	0	0		0	0			0	0	0	0	0	0	0	0	0
Hybrid						1			2	7	0		0			0	0		0	2	0		3	0			0	1	13	2	6	0	0	0	0
Fhybrid						0			1	11	0		0			0	0		0	1	0		0	0			0	0	1	0	0	1	0	0	0
HexNAc(3)(x)						2			3	11	0		0			0	6		2	3	1		11	0			0	3	7	6	22	0	0	0	0
HexNAc(3)(F)(x)						0			0	10	3		0			0	1		12	2	0		0	2			0	0	1	5	2	5	0	0	0
HexNAc(4)(x)						0			1	1	0		0			0	1		0	3	1		1	0			0	0	2	4	27	1	0	0	0
HexNAc(4)(F)(x)						0			7	6	33		0			0	20		64	17	11		8	31			0	0	5	25	2	26	0	0	0
HexNAc(5)(x)						2			1	0	0		0			0	18		0	2	0		0	0			0	0	0	1	20	0	0	0	0
HexNAc(5)(F)(x)						0			4	1	60		0			0	49		15	3	0		21	63			0	0	1	14	0	25	0	0	0
HexNAc(6+)(x)						0			0	0	0		0			0	0		0	0	0		0	0			0	0	0	0	1	5	0	0	0
HexNAc(6+)(F)(x)						0			0	0	4		0			0	1		0	0	0		0	3			0	0	0	5	0	19	100	0	100
Unoccupied						0			15	0	0		0			0	1		0	1	0		0	0			0	0	1	4	1	16	0	0	0
Core						0			1	0	0		2			1	0		1	0	0		0	0			0	0	0	0	0	0	0	0	0
Oligomannose						93			65	53	0		98			98	2		6	64	87		55	1			100	95	69	33	20	2	0		0
Hybrid						1			3	18	0		0			0	0		0	2	0		3	0			0	1	14	2	6	1	0	0	0
Complex						6			17	29	100		2			1	97		94	32	13		42	99			0	3	17	61	73	82	100	0	100
Unoccupied						0			15	0	0		0			0	1		0	1	0		0	0			0	0	1	4	1	16	0	0	0
Fucose						0			12	28	100		0			0	71		91	23	12		30	99			0	0	7	49	4	77	100	0	100
NeuAc						0			2	6	26		0			0	8		18	6	1		0	11			0	0	8	13	34	14	76	0	75

WIV1	N17	N20	N28	N30	N59	N61	N69	N74	N112	N122	N149	N162	N165	N176	N185	N234	N282	N307	N331	N343	N370	N554	N603	N616	N657	N677	N709	N717	N801	N1074	N1098	N1134	N1158	N1173	N1194
M9Glc						0			0	0	0		0			0	0		0	0	0		0	0			0	0	0	0	0	0	0	0	0
M9						0			0	0	0		0			45	0		0	0	0		0	0			0	0	0	0	0	0	0	0	0
M8						0			0	0	0		0			40	0		0	0	0		0	0			0	0	0	0	0	0	0	0	0
M7						0			0	0	0		6			6	0		0	0	0		0	0			0	13	37	0	0	0	0	0	0
M6						0			0	2	0		21			3	0		0	0	2		0	0			0	24	5	0	0	0	0	0	0
M5						26			2	31	0		65			4	1		5	4	61		56	1			93	26	35	12	13	1	0	0	0
M4						0			0	0	0		0			0	0		0	1	0		3	0			7	3	0	5	0	0	0	0	0
M3						0			0	0	0		2			0	0		0	0	0		0	0			0	0	0	2	0	0	0	0	0
FM						0			0	0	0		0			0	0		1	0	0		0	0			0	0	0	0	0	0	0	0	0
Hybrid						0			0	2	0		3			0	0		0	0	9		3	0			0	0	7	1	12	0	0	0	0
Fhybrid						0			0	9	0		1			0	0		1	1	4		0	0			0	0	0	0	0	0	0	0	0
HexNAc(3)(x)						3			0	9	0		0			0	1		2	0	0		7	0			0	0	4	4	19	1	0	0	0
HexNAc(3)(F)(x)						0			1	22	4		0			0	0		10	2	3		0	0			0	0	0	3	1	3	0	0	0
HexNAc(4)(x)						28			3	2	0		0			0	10		0	0	4		0	0			0	0	0	2	31	0	0	0	0
HexNAc(4)(F)(x)						0			42	21	22		1			0	42		55	69	16		3	31			0	0	7	27	0	20	0	0	0
HexNAc(5)(x)						37			3	1	0		0			0	8		0	0	0		0	0			0	0	0	1	18	0	0	0	0
HexNAc(5)(F)(x)						1			26	1	75		0			0	33		22	23	1		28	66			0	0	1	35	0	15	0	0	0
HexNAc(6+)(x)						1			0	0	0		0			0	0		0	0	0		0	0			0	0	0	0	6	0	0	0	0
HexNAc(6+)(F)(x)						0			0	0	0		0			0	5		0	0	0		0	2			0	0	0	2	0	6	100	0	0
Unoccupied						0			23	0	0		0			0	0		0	0	0		0	0			0	13	0	4	0	55	0	0	100
Core						3			0	0	0		1			1	0		4	0	0		0	0			0	21	4	1	0	0	0	0	0
Oligomannose						26			2	33	0		92			99	1		6	5	63		59	1			100	65	78	17	14	1	0		0
Hybrid						0			0	11	0		4			0	0		1	1	13		3	0			0	0	7	1	12	0	0	0	0
Complex						74			75	56	100		4			1	99		93	94	24		38	99			0	21	15	78	74	44	100	0	0
Unoccupied						0			23	0	0		0			0	0		0	0	0		0	0			0	13	0	4	0	55	0	0	100
Fucose						2			70	53	100		2			0	80		88	95	24		31	99			0	0	8	68	1	43	100	0	0
NeuAc						12			22	7	47		0			0	16		19	52	28		0	13			0	0	8	7	52	1	65	0	0

SARS CoV 1	N17	N20	N28	N30	N59	N61	N69	N74	N112	N122	N149	N162	N165	N176	N185	N234	N282	N307	N331	N343	N370	N554	N603	N616	N657	N677	N709	N717	N801	N1074	N1098	N1134	N1158	N1173	N1194
M9Glc				0		0	0		0	0			0			0	0		0	0	0		0	0			0	0	0	0	0	0	0	0	0
M9				0		0	0		0	0			0			0	0		0	0	0		0	0			0	0	0	0	0	0	0	0	0
M8				0		1	0		0	0			0			50	0		0	0	0		0	0			0	0	0	0	0	0	0	0	0
M7				0		8	0		0	0			0			12	0		0	0	0		0	0			0	16	0	0	0	0	0	0	0
M6				0		38	0		0	0			13			23	0		0	0	0		0	0			0	19	43	0	2	0	0	0	0
M5				0		39	9		1	0			69			12	0		9	20	17		42	0			0	38	2	0	25	0	0	0	0
M4				0		0	0		0	0			0			0	0		0	0	0		0	0			0	0	0	0	0	0	0	0	0
M3				0		0	0		0	0			0			0	0		0	0	0		0	0			0	0	0	0	0	0	0	0	0
FM				0		0	0		0	0			0			0	0		0	0	0		0	0			0	0	0	0	0	0	0	0	0
Hybrid				0		5	0		0	0			14			0	0		1	1	4		0	0			0	9	18	4	12	0	0	0	0
Fhybrid				0		0	0		0	0			0			0	0		1	1	9		0	2			0	2	0	3	0	0	0	0	0
HexNAc(3)(x)				0		9	0		0	1			0			0	2		2	2	2		0	6			0	5	1	3	26	0	0	0	0
HexNAc(3)(F)(x)				0		0	0		2	4			0			0	0		12	6	16		12	17			0	3	4	7	0	7	0	0	0
HexNAc(4)(x)				0		1	0		7	0			0			0	0		0	0	4		0	2			0	0	30	5	28	0	0	0	0
HexNAc(4)(F)(x)				0		0	41		64	72			0			0	47		57	65	45		34	23			0	7	2	65	2	85	0	0	0
HexNAc(5)(x)				0		0	0		2	0			0			0	4		0	0	0		0	15			0	0	0	0	4	0	0	0	0
HexNAc(5)(F)(x)				14		0	29		22	21			3			0	45		17	3	2		13	33			0	1	0	8	0	0	0	0	89
HexNAc(6+)(x)				0		0	0		0	0			0			0	1		0	0	0		0	0			0	0	0	0	0	0	0	0	0
HexNAc(6+)(F)(x)				0		0	5		3	2			0			0	2		1	0	0		0	2			0	0	0	3	0	8	0	0	11
Unoccupied				86		0	16		0	0			0			2	0		0	0	0		0	0			0	0	0	0	0	0	0	0	0
Core				0		0	0		0	0			0			0	0		0	0	0		0	0			0	0	0	0	0	0	0	0	0
Oligomannose				0		86	9		1	0			83			97	0		10	21	17		42	0				73	45	0	28	0	0	0	0
Hybrid				0		5	0		0	0			14			0	0		1	2	14		0	2			0	10	18	7	12	0	0	0	0
Complex				14		10	75		99	100			3			0	100		89	77	69		58	98			0	16	37	92	61	100	0	0	100
Unoccupied				86		0	16		0	0			0			2	0		0	0	0		0	0			0	0	0	0	0	0	0	0	0

Supplemental Table 5: Site-specific glycan analysis of clade 1b sarbecoviruses (related to Figure 2 and 3)

Pang17	N17	N20	N28	N30	N59	N61	N69	N74	N112	N122	N149	N162	N165	N176	N185	N234	N282	N307	N331	N343	N370	N554	N603	N616	N657	N677	N709	N717	N801	N1074	N1098	N1134	N1158	N1173	N1194
M9Glc	0			0		0				0	0		0			0	0		0	0	0		0	0	0		0	0	0	0	0	0	0	0	0
M9	100			0		0				0	0		0			60	0		0	0	1		0	0	0		0	0	0	0	0	0	0	0	0
M8	0			1		1				0	0		0			28	0		0	0	4		0	0	0		0	0	1	0	0	0	0	0	0
M7	0			16		1				4	0		0			6	0		0	0	14		6	0	0		0	8	6	0	1	0	0	0	0
M6	0			52		5				4	0		5			3	0		0	1	31		5	0	0		0	7	3	0	0	0	0	0	0
M5	0			26		62				55	4		88			2	15		3	64	48		56	16	0		100	79	66	57	5	7	0	0	0
M4	0			3		9				1	0		1			0	0		0	0	1		27	0	0		0	0	5	1	0	0	0	0	0
M3	0			0		2				0	0		0			0	0		0	0	0		2	0	0		0	0	2	0	0	0	0	0	0
FM	0			0		0				0	0		0			0	0		0	0	0		0	1	0		0	0	0	0	0	0	0	0	0
Hybrid	0			0		9				15	0		1			0	4		0	2	0		0	9	0		0	0	4	3	19	1	0	0	0
Fhybrid	0			0		2				3	0		0			0	0		0	3	0		0	4	0		0	0	0	3	4	5	0	0	0
HexNAc(3)(x)	0			0		2				11	0		2			0	9		1	1	1		2	22	0		0	6	4	7	23	0	0	0	0
HexNAc(3)(F)(x)	0			0		0				3	5		0			0	11		7	10	0		0	7	0		0	0	0	9	3	20	0	0	0
HexNAc(4)(x)	0			0		2				2	0		0			0	0		0	0	0		0	13	0		0	0	0	1	15	0	0	0	0
HexNAc(4)(F)(x)	0			0		0				2	40		3			0	44		67	15	0		2	23	79		0	0	0	10	16	46	0	0	0
HexNAc(5)(x)	0			0		1				0	0		0			0	0		0	0	0		0	1	0		0	0	0	0	3	0	0	0	0
HexNAc(5)(F)(x)	0			0		0				0	50		0			0	17		21	4	0		0	3	0		0	0	0	8	6	17	0	0	0
HexNAc(6+)(x)	0			0		0				0	0		0			0	0		0	0	0		0	0	0		0	0	0	0	1	0	0	0	0
HexNAc(6+)(F)(x)	0			0		0				0	0		0			0	0		0	0	0		0	0	0		0	0	0	0	0	0	100	0	100
Unoccupied	0			1		1				0	1		0			1	0		0	0	0		0	0	21		0	0	0	1	3	4	0	100	0
Core	0			1		3				0	0		0			0	0		0	0	0		0	0	0		0	0	8	0	0	0	0	0	0
Oligomannose	100			98		78				63	4		94			98	15		4	66	99		94	17	0		100	94	81	58	6	7	0	0	0
Hybrid	0			0		11				18	0		1			0	4		0	5	0		0	13	0		0	0	4	5	24	6	0	0	0
Complex	0			2		10				19	96		5			0	81		96	30	1		6	70	79		0	6	14	35	67	83	100	0	100
Unoccupied	0			1		1				0	1		0			1	0		0	0	0		0	0	21		0	0	0	1	3	4	0	100	0
Fucose	0			0		2				7	95		3			0	71		96	31	0		2	38	79		0	0	0	30	29	88	100	0	100
NeuAc	0			0		1				8	24		1			0	4		23	1	0		0	0	0		0	0	1	4	46	7	58	0	44

RaTG13	N17	N20	N28	N30	N59	N61	N69	N74	N112	N122	N149	N162	N165	N176	N185	N234	N282	N307	N331	N343	N370	N554	N603	N616	N657	N677	N709	N717	N801	N1074	N1098	N1134	N1158	N1173	N1194
M9Glc	0			0		0				0	0		0			0	0		0	0	0		0	0	0		0	0	0	0	0	0	0	0	0
M9	0			1		0				0	0		0			14	0		0	0	0		0	0	0		0	0	0	0	0	0	0	0	0
M8	0			4		1				0	0		0			29	0		0	0	0		0	0	0		0	0	0	0	0	0	0	0	0
M7	0			28		1				0	0		0			17	0		0	0	0		0	0	0		0	0	0	0	0	0	0	0	0
M6	0			45		13				0	0		0			11	0		0	0	0		0	0	0		0	2	0	0	0	0	0	0	0
M5	0			16		63				17	0		5			19	0		1	11	7		1	0	0		0	34	15	2	1	0	0	0	0
M4	0			1		3				1	0		0			1	0		0	0	0		0	0	0		0	3	0	0	0	0	0	0	0
M3	0			0		0				0	0		0			0	0		0	0	0		0	0	0		0	0	1	0	0	0	0	0	0
FM	0			0		0				0	0		0			0	0		0	0	0		0	0	0		0	0	0	0	0	0	0	0	0
Hybrid	0			0		1				5	0		0			3	0		0	0	0		0	0	0		0	10	2	0	0	0	0	0	0
Fhybrid	0			0		0				1	0		0			0	0		0	0	7		0	0	0		0	7	0	2	0	0	0	0	0
HexNAc(3)(x)	0			0		1				5	0		0			2	0		0	0	0		0	0	0		0	0	3	1	2	0	0	0	0
HexNAc(3)(F)(x)	0			0		0				1	4		0			1	0		5	4	12		1	2	0		0	20	2	1	1	1	0	0	0
HexNAc(4)(x)	0			0		0				31	0		0			0	0		0	0	2		0	0	0		0	0	7	0	14	0	0	0	0
HexNAc(4)(F)(x)	0			0		0				20	41		51			1	24		62	71	72		56	75	49		100	6	33	27	17	28	0	0	0
HexNAc(5)(x)	0			0		0				12	0		0			0	0		0	0	0		0	0	0		0	0	1	0	11	5	0	0	0
HexNAc(5)(F)(x)	0			0		0				6	50		34			0	56		32	12	1		25	23	5		0	2	21	37	14	26	0	0	0
HexNAc(6+)(x)	0			0		0				0	0		0			0	0		0	0	0		0	0	0		0	0	0	0	17	13	0	0	0
HexNAc(6+)(F)(x)	0			0		0				0	0		10			0	20		0	0	0		0	0	0		0	0	1	28	19	26	3	0	13
Unoccupied	100			4		16				0	4		0			1	0		0	0	0		17	0	46		0	17	10	1	4	2	97	100	87
Core	0			1		1				0	0		0			0	0		0	1	0		0	0	0		0	0	4	0	0	0	0	0	0
Oligomannose	0			95		81				19	0		5			91	0		1	11	7		1	0	0		0	39	16	2	1	0	0	0	0
Hybrid	0			0		1				7	0		0			3	0		0	0	7		0	0	0		0	17	2	2	0	0	0	0	0
Complex	0			1		2				75	96		95			5	100		99	88	86		82	100	54		100	28	73	94	96	98	3	0	13
Unoccupied	100			4		16				0	4		0			1	0		0	0	0		17	0	46		0	17	10	1	4	2	97	100	87
Fucose	0			0		0				28	96		95			3	100		99	87	92		82	100	54		100	35	57	95	51	80	3	0	13
NeuAc	0			0		0				15	42		0			0	13		28	9	26		5	13	17		0	2	3	25	72	15	3	0	13

SARS CoV 2	N17	N20	N28	N30	N59	N61	N69	N74	N112	N122	N149	N162	N165	N176	N185	N234	N282	N307	N331	N343	N370	N554	N603	N616	N657	N677	N709	N717	N801	N1074	N1098	N1134	N1158	N1173	N1194
M9Glc	0					0		0		0	0		0			0	0		0	0			0	0	0		0	0	0	0	0	0	0	0	0
M9	0					0		0		0	0		0			22	0		0	0			0	0	0		0	1	1	0	1	0	0	0	0
M8	0					2		0		0	0		0			28	0		0	0			1	0	0		3	1	2	1	1	0	0	0	0
M7	0					2		0		3	0		0			15	0		0	0			2	0	0		8	14	5	1	2	0	0	0	0
M6	0					6		0		1	1		1			5	0		0	0			3	2	0		12	13	3	2	1	0	0	0	0
M5	4					26		1		12	3		17			7	3		4	2			17	2	3		26	17	16	14	4	3	13	7	6
M4	0					2		0		0	2		0			1	0		0	0			2	0	0		0	3	3	0	0	0	0	0	0
M3	0					1		0		0	0		0			0	0		0	0			0	0	0		0	0	0	0	0	0	0	0	0
FM	0					0		1		0	0		0			0	0		0	0			0	0	0		0	0	0	0	0	0	0	0	0
Hybrid	1					11		0		13	0		2			4	1		1	0			7	4	0		0	22	17	5	8	1	0	0	0
Fhybrid	2					1		1		3	1		1			1	1		1	1			4	3	1		4	6	1	5	2	2	0	2	0
HexNAc(3)(x)	0					5		0		7	0		1			1	1		0	0			5	4	0		0	7	4	3	3	0	0	0	1
HexNAc(3)(F)(x)	4					0		4		4	4		0			0	1		2	2			2	4	1		4	3	1	6	2	2	0	4	7
HexNAc(4)(x)	0					18		0		25	2		5			7	4		4	1			1	8	0		0	3	14	3	24	0	0	0	0
HexNAc(4)(F)(x)	40					9		30		18	43		32			1	30		52	58			34	39	43		24	8	24	24	15	39	16	15	16
HexNAc(5)(x)	0					11		0		5	1		4			0	10		0	0			0	6	1		0	0	2	1	13	0	0	0	13
HexNAc(5)(F)(x)	37					2		44		6	36		33			0	38		31	34			22	21	33		16	2	6	24	12	20	23	9	3
HexNAc(6+)(x)	0					1		0		0	0		0			6	1		0	0			0	0	0		0	0	0	0	8	0	0	0	0
HexNAc(6+)(F)(x)	5					2		19		0	6		3			0	10		4	1			0	5	12		3	0	0	8	5	15	47	27	18
Unoccupied	6					0		0		1	1		0			1	0		0	0			0	0	6		0	0	0	2	0	15	0	35	36
Core	0					1		0		0	0		0			0	0		0	0			0	0	0		0	0	1	0	0	0	0	1	0
Oligomannose	5					38		2		17	6		19			80	4	0	4	2			25	4	3		49	48	30	19	7	5	13	7	7
Hybrid	3					11		1		16	1		2			4	1	0	2	1			11	8	1		4	28	18	10	10	3	0	2	0
Complex	86					51		97		66	92		79			15	95	0	94	97			64	88	90		47	24	52	69	82	77	87	56	57
Unoccupied	6					0		0		1	1		0			1	0	0	0	0			0	0	6		0	0	0	2	0	15	0	35	36
Fucose	87					14		98		32	90		69			3	79		91	96			63	73	89		50	19	32	67	36	78	86	57	43
NeuAc	26					7		43		21	35		18			0	15		31	37			4	9	33		6	6	28	13	46	18	41	14	32

Supplemental Table 6: Site-specific glycan analysis of clade 2 sarbecoviruses (related to Figure 2 and 3)

Yun11	N17	N20	N28	N30	N59	N61	N69	N74	N112	N122	N149	N162	N165	N176	N185	N234	N282	N307	N331	N343	N370	N554	N603	N616	N657	N677	N709	N717	N801	N1074	N1098	N1134	N1158	N1173	N1194
M9Glc						0			0	0			0			0	0	0	0		0		0	0	0		0	0	0	0	0	0	0	0	0
M9						0			0	0			0			13	0	0	0		0		0	0	0		0	0	0	0	0	0	0	0	0
M8						0			0	0			0			41	0	0	0		0		0	0	0		0	0	0	0	0	0	0	0	0
M7						3			0	0			0			19	0	1	0		0		0	0	0		0	0	1	0	0	0	0	0	0
M6						60			0	0			7			11	0	6	0		1		2	0	0		0	16	0	0	0	0	0	0	0
M5						33			0	0			53			7	2	39	1		14		17	0	0		0	52	16	2	2	1	0	0	0
M4						0			0	0			2			1	0	11	0		0		1	0	0		0	0	3	1	0	0	0	0	0
M3						3			0	0			0			0	0	6	0		0		0	0	0		0	0	1	0	0	0	0	0	0
FM						0			0	0			0			0	0	0	0		0		0	0	0		0	0	0	0	0	0	0	0	0
Hybrid						0			0	0			12			0	0	5	0		0		1	0	0		0	8	4	0	0	0	0	0	0
Fhybrid						0			0	0			5			0	0	0	0		0		3	2	0		0	2	0	3	0	0	0	0	0
HexNAc(3)(x)						0			0	0			1			0	0	7	0		1		3	0	0		0	2	3	1	2	0	0	0	0
HexNAc(3)(F)(x)						0			0	0			3			0	0	0	4		0		9	8	0		0	5	0	9	2	2	0	0	0
HexNAc(4)(x)						0			0	0			0			2	0	4	0		0		0	0	0		0	0	2	0	9	0	0	0	0
HexNAc(4)(F)(x)						0			0	0			12			0	26	2	57		70		64	71	70		0	0	52	45	55	25	0	0	0
HexNAc(5)(x)						0			0	100			0			0	0	0	0		0		0	0	0		0	0	0	0	0	0	0	0	0
HexNAc(5)(F)(x)						0			0	0			4			0	68	0	31		15		0	20	18		0	0	18	36	26	12	0	0	0
HexNAc(6+)(x)						0			0	0			0			0	0	0	0		0		0	0	0		0	0	0	0	0	0	0	0	0
HexNAc(6+)(F)(x)						0			0	0			0			0	4	0	2		0		0	0	0		0	0	0	2	3	0	0	0	100
Unoccupied						0			0	0			0			2	0	7	4		0		0	0	8		0	0	0	1	1	61	0	100	0
Core						1			0	0			0			4	0	13	1		0		0	0	4		0	15	0	1	0	0	0	0	0
Oligomannose						95			0	0			63			91	2	57	1		15		20	0	0			67	19	3	2	1	0	0	0
Hybrid						0			0	0			17			0	0	5	0		0		4	2	0		0	10	4	3	0	0	0	0	0
Complex						4			0	100			20			7	98	31	94		85		76	98	92		0	23	77	93	96	39	0	0	100
Unoccupied						0			0	0			0			2	0	7	4		0		0	0	8		0	0	0	1	1	61	0	100	0
Fucose						0			0	0			24			0	98	2	94		84		76	100	88		0	7	70	94	86	39	0	0	100
NeuAc						0			0	0			6			2	0	0	30		32		0	0	33		0	1	28	19	81	3	0	0	64

Rf1	N17	N20	N28	N30	N59	N61	N69	N74	N112	N122	N149	N162	N165	N176	N185	N234	N282	N307	N331	N343	N370	N554	N603	N616	N657	N677	N709	N717	N801	N1074	N1098	N1134	N1158	N1173	N1194
M9Glc						0			0	0			0		0	0	0	0	0	0	0		0	0	0		0	0	0	0	0	0	0	0	0
M9						0			0	0			0		0	0	0	0	0	0	0		0	0	0		0	0	0	0	0	0	0	0	0
M8						2			0	0			0		0	12	0	0	0	0	0		0	0	0		0	0	0	0	0	0	0	0	0
M7						2			0	0			0		0	18	0	0	0	0	0		0	0	0		0	1	0	0	0	0	0	0	0
M6						33			0	0			0		0	14	0	5	0	0	0		0	0	0		0	1	0	0	0	0	0	0	0
M5						44			0	100			20		5	28	5	90	8	9	0		44	4	5		100	83	24	9	12	5	8	100	7
M4						3			0	0			0		1	3	0	0	0	2	0		6	0	0		0	0	1	0	0	0	0	0	0
M3						0			0	0			0		0	0	0	0	0	0	0		2	0	0		0	0	0	0	0	0	0	0	0
FM						0			0	0			0		1	0	0	0	1	0	0		0	0	1		0	0	0	0	0	0	0	0	0
Hybrid						0			0	0			0		0	0	0	0	0	0	0		2	0	0		0	3	2	1	0	0	0	0	0
Fhybrid						0			0	0			0		0	0	0	0	0	0	1		0	1	0		0	1	1	3	0	0	0	0	0
HexNAc(3)(x)						1			0	0			1		0	2	0	5	1	2	0		9	2	1		0	0	5	5	1	0	0	0	0
HexNAc(3)(F)(x)						0			0	0			0		6	0	1	0	8	9	11		18	2	7		0	10	9	22	5	1	4	0	0
HexNAc(4)(x)						0			0	0			0		0	0	0	0	0	0	0		0	0	0		0	0	1	1	14	0	0	0	0
HexNAc(4)(F)(x)						0			0	0			60		58	0	60	0	49	65	85		18	88	70		0	1	38	37	60	62	9	0	0
HexNAc(5)(x)						0			0	0			0		0	0	0	0	0	0	0		0	0	0		0	0	1	0	0	0	0	0	0
HexNAc(5)(F)(x)						0			0	0			19		22	0	30	0	23	10	2		0	2	10		0	0	14	15	2	20	26	0	0
HexNAc(6+)(x)						0			0	0			0		0	0	0	0	0	0	0		0	0	0		0	0	0	0	0	0	0	0	0
HexNAc(6+)(F)(x)						0			0	0			0		1	0	0	0	1	0	0		0	0	0		0	0	0	2	0	9	54	0	0
Unoccupied						0			0	0			0		0	23	0	0	0	0	0		0	1	2		0	0	0	3	6	3	0	0	93
Core						15			0	0			0		7	0	3	0	9	2	0		1	0	5		0	0	3	1	0	0	0	0	0
Oligomannose						85				100			20		7	75	5	95	9	11	0		50	4	6		100	85	26	9	12	5	8	100	7
Hybrid						0			0	0			0		0	0	0	0	0	0	1		2	1	0		0	4	2	4	0	0	0	0	0
Complex						15			0	0			80		93	2	95	5	91	88	99		48	94	92		0	11	71	83	82	92	92	0	0
Unoccupied						0			0	0			0		0	23	0	0	0	0	0		0	1	2		0	0	0	3	6	3	0	0	93
Fucose						0			0	0			79		87	0	92	0	81	85	100		36	93	87		0	12	62	79	67	92	92	0	0
NeuAc						0			0	0			6		19	0	4	0	9	4	11		0	11	23		0	0	9	6	35	0	23	0	0

RmYN02	N17	N20	N28	N30	N59	N61	N69	N74	N112	N122	N149	N162	N165	N176	N185	N234	N282	N307	N331	N343	N370	N554	N603	N616	N657	N677	N709	N717	N801	N1074	N1098	N1134	N1158	N1173	N1194
M9Glc			0		0				0	0			0			0	0	0	0	0	0			0	0		0	0	0	0	0	0	0	0	0
M9			0		38				0	0			0			16	0	0	0	0	0			0	0		0	0	0	0	0	0	0	0	0
M8			0		24				0	0			0			27	0	2	0	0	0			0	0		0	0	0	0	0	0	0	0	0
M7			0		9				0	0			0			13	0	3	0	0	1			0	0		0	4	0	0	0	0	0	0	0
M6			0		5				0	0			0			10	0	2	0	0	3			0	0		0	6	0	0	1	0	0	0	0
M5			33		7				4	6			39			12	11	22	5	8	16			2	0		5	24	11	11	6	3	0	0	0
M4			1		2				0	0			4			4	0	1	0	1	0			0	0		0	2	1	3	0	0	0	0	0
M3			0		0				0	0			0			1	0	1	0	0	0			0	0		0	1	0	0	0	0	0	0	0
FM			0		0				0	0			0			0	0	0	0	0	0			0	0		0	0	0	1	0	0	0	0	0
Hybrid			1		0				0	0			0			1	0	1	0	0	3			0	0		0	19	2	1	4	0	0	0	0
Fhybrid			0		0				0	0			0			0	0	0	0	0	0			0	0		0	2	0	3	0	0	0	0	0
HexNAc(3)(x)			4		0				0	0			0			0	2	8	0	1	9			0	0		0	16	7	5	18	0	0	0	0
HexNAc(3)(F)(x)			1		0				0	0			0			0	0	2	3	0	0			0	0		0	2	2	16	7	2	0	0	0
HexNAc(4)(x)			9		0				0	0			0			0	0	21	0	5	0			0	0		0	0	5	0	11	0	0	0	0
HexNAc(4)(F)(x)			15		0				25	55			19			0	14	16	51	65	63			24	20		0	5	33	19	12	23	0	0	0
HexNAc(5)(x)			1		0				0	0			0			0	1	2	0	1	0			0	0		0	0	1	0	8	0	0	0	0
HexNAc(5)(F)(x)			10		0				46	26			37			0	50	2	32	17	4			38	4		0	1	8	21	6	26	0	0	0
HexNAc(6+)(x)			0		0				0	0			0			0	0	0	0	0	0			0	0		0	0	0	0	11	0	0	0	0
HexNAc(6+)(F)(x)			0		0				0	13			0			0	2	0	9	0	0			36	9		0	0	0	15	4	45	2	0	6
Unoccupied			25		10				25	0			0			15	20	13	0	0	0			0	68		95	16	28	5	13	1	98	0	94
Core			1		5				0	0			0			0	0	3	0	0	0			0	0		0	1	1	1	0	0	0	0	0
Oligomannose			34		85				4	6			43			82	11	31	5	10	20			2	0		5	36	12	14	7	3	0		0
Hybrid			1		0				0	0			0			1	0	1	0	0	3			0	0		0	21	3	3	4	0	0	0	0
Complex			40		5				71	94			57			2	69	55	95	90	77			98	32		0	27	57	77	77	96	2	0	6
Unoccupied			25		10				25	0			0			15	20	13	0	0	0			0	68		95	16	28	5	13	1	98	0	94
Fucose			27		0				71	94			57			0	66	19	95	82	67			98	32		0	10	43	75	30	96	2	0	6
NeuAc			8		0				5	21			0			0	8	0	20	13	20			0	24		0	3	10	2	39	0	0	0	6

RS4081	N17	N20	N28	N30	N59	N61	N69	N74	N112	N122	N149	N162	N165	N176	N185	N234	N282	N307	N331	N343	N370	N554	N603	N616	N657	N677	N709	N717	N801	N1074	N1098	N1134	N1158	N1173	N1194
M9Glc						0			0	0			0			0	0	0	0	0	0		0	0	0		0	0	0	0	0	0	0	0	0
M9						0			0	0			0			2	0	0	0	0	0		0	0	0		0	0	0	0	0	0	0	0	0
M8						0			0	0			0			17	0	0	0	0	0		0	0	0		0	0	0	0	0	0	0	0	0
M7						0			0	0			0			16	0	38	0	0	1		0	0	0		0	7	1	0	0	0	0	0	0
M6						2			0	0			0			11	0	25	0	0	1		1	0	0		0	16	1	0	0	0	0	0	0
M5						61			0	0			12			20	0	37	3	19	26		19	0	0		0	64	25	4	3	1	0	0	0
M4						4			0	0			0			5	0	0	0	5	0		1	0	0		0	6	2	0	0	0	0	0	0
M3						0			0	0			0			1	0	0	0	0	0		0	0	0		0	0	1	0	0	0	0	0	0
FM						0			0	0			0			0	0	0	0	0	0		0	0	0		0	0	0	0	0	0	0	0	0
Hybrid						5			0	0			0			2	0	0	0	1	1		0	0	0		0	2	6	1	1	0	0	0	0
Fhybrid						1			0	0			1			0	0	0	0	0	3		0	1	0		0	0	0	2	1	0	0	0	0
HexNAc(3)(x)						7			0	0			2			1	0	0	0	1	1		3	0	0		0	4	4	2	5	0	0	0	0
HexNAc(3)(F)(x)						1			0	0			3			1	0	0	5	5	4		12	6	0		0	0	3	12	2	0	0	0	0
HexNAc(4)(x)						10			0	0			0			0	0	0	0	1	0		0	1	0		0	0	4	2	24	0	0	0	0
HexNAc(4)(F)(x)						3			0	0			59			1	41	0	56	46	60		63	70	56		0	0	39	51	44	20	0	0	0
HexNAc(5)(x)						6			0	0			0			0	0	0	0	1	0		0	0	0		0	0	1	0	6	0	0	0	0
HexNAc(5)(F)(x)						1			0	0			24			0	59	0	29	17	2		0	23	12		0	0	12	18	10	2	0	0	0
HexNAc(6+)(x)						0			0	0			0			0	0	0	0	0	0		0	0	0		0	0	0	0	0	0	0	0	0
HexNAc(6+)(F)(x)						0			0	0			0			0	0	0	2	0	0		0	0	0		0	0	0	2	2	0	100	0	0
Unoccupied						0			0	0			0			21	0	0	4	2	0		0	0	33		0	0	0	5	0	77	0	0	0
Core						0			0	0			0			2	0	0	0	1	0		0	0	0		0	0	1	0	2	0	0	0	0
Oligomannose						67							12			71	0	100	3	24	29		22	0	0			94	29	5	3	1	0		
Hybrid						6			0	0			1			2	0	0	0	1	4		0	1	0		0	2	6	4	2	0	0	0	0
Complex						26			0	0			87			6	100	0	92	73	67		78	99	67		0	4	64	86	95	22	100	0	0
Unoccupied						0			0	0			0			21	0	0	4	2	0		0	0	33		0	0	0	5	0	77	0	0	0
Fucose						5			0	0			87			2	100	0	92	68	69		74	99	67		0	0	55	85	59	22	100	0	0
NeuAc						3			0	0			7			0	6	0	22	1	19		0	5	16		0	2	22	13	64	2	60	0	0

Supplemental Table 7: Site-specific glycan analysis of clade 3 sarbecoviruses (related to Figure 2 and 3)

BtKY72	N17	N20	N28	N30	N59	N61	N69	N74	N112	N122	N149	N162	N165	N176	N185	N234	N282	N307	N331	N343	N370	N554	N603	N616	N657	N677	N709	N717	N801	N1074	N1098	N1134	N1158	N1173	N1194
M9Glc		0				0			0	0	0	0	0			0	0		0	0	0		0	0	0			0	0	0	0	0	0	0	0
M9		0				0			0	0	0	0	0			6	0		0	0	0		0	0	0			0	0	0	0	0	0	0	0
M8		0				0			0	0	0	0	0			26	0		0	0	0		0	0	0			0	0	0	0	0	0	0	0
M7		0				0			0	0	0	0	0			27	0		0	0	0		0	0	0			0	0	0	0	0	0	0	0
M6		0				0			0	0	0	0	0			18	0		0	0	0		0	0	0			0	0	0	2	0	0	0	0
M5		3				37			64	46	1	7	0			22	7		5	3	16		46	3	12			19	38	7	21	8	0	0	18
M4		0				0			2	0	0	0	0			0	0		0	0	0		0	0	0			4	1	0	0	0	0	0	0
M3		0				0			0	5	0	0	0			0	0		0	0	0		0	0	0			2	0	0	0	0	0	0	0
FM		0				0			0	0	0	0	0			0	0		0	0	0		0	0	0			2	0	0	0	0	0	0	0
Hybrid		0				1			0	1	0	0	0			0	0		0	0	0		0	0	0			9	2	0	0	0	0	0	0
Fhybrid		0				0			0	0	0	0	0			0	0		0	0	0		0	0	0			4	1	0	0	0	0	0	0
HexNAc(3)(x)		0				11			12	6	0	0	0			1	0		1	0	0		0	0	0			18	5	0	0	0	0	0	0
HexNAc(3)(F)(x)		0				0			0	0	0	0	0			0	0		5	2	0		0	0	0			28	6	0	0	0	0	0	0
HexNAc(4)(x)		0				33			11	21	0	0	0			0	0		0	0	0		0	0	0			0	1	0	3	0	0	0	0
HexNAc(4)(F)(x)		27				6			12	1	3	52	0			0	0		51	62	84		54	31	29			7	25	37	9	7	0	0	0
HexNAc(5)(x)		0				11			0	7	0	0	0			0	0		0	0	0		0	0	0			0	1	0	0	0	0	0	0
HexNAc(5)(F)(x)		8				0			0	0	5	41	0			0	0		35	32	0		0	21	27			3	16	0	0	0	0	0	0
HexNAc(6+)(x)		0				0			0	0	0	0	0			0	0		0	0	0		0	0	0			0	0	0	0	0	0	0	0
HexNAc(6+)(F)(x)		0				0			0	0	1	0	0			0	22		3	0	0		0	46	32			3	0	56	33	0	100	0	15
Unoccupied		59				1			0	12	89	0	0			0	70		0	0	0		0	0	0			0	0	0	32	86	0	0	67
Core		4				0			0	0	0	0	0			0	0		0	0	0		0	0	0			1	3	0	0	0	0	0	0
Oligomannose		3				37			66	46	1	7	0			99	7		5	4	16		46	3	12			24	39	7	23	8	0		18
Hybrid		0				1			0	1	0	0	0			0	0		0	0	0		0	0	0			13	4	0	0	0	0	0	0
Complex		38				61			34	40	10	93	0			1	22		95	96	84		54	97	88			62	57	93	44	7	100	0	15
Unoccupied		59				1			0	12	89	0	0			0	70		0	0	0		0	0	0			0	0	0	32	86	0	0	67
Fucose		35				6			12	1	10	93	0			0	22		94	96	84		54	97	88			47	48	93	41	7	100	0	15
NeuAc		20				13			0	10	1	36	0			0	0		26	12	27		0	2	4			7	7	27	24	0	42	0	0

BM4831	N17	N20	N28	N30	N59	N61	N69	N74	N112	N122	N149	N162	N165	N176	N185	N234	N282	N307	N331	N343	N370	N554	N603	N616	N657	N677	N709	N717	N801	N1074	N1098	N1134	N1158	N1173	N1194
M9Glc		0				0			0	0			0	0		0	0		0	0	0	0	0	0	0	0	0	0	0	0	0	0	0	0	0
M9		0				0			0	0			0	0		16	0		0	0	0	0	0	0	0	0	0	0	0	0	0	0	0	0	0
M8		0				6			0	0			0	0		32	0		0	0	0	0	0	0	0	0	0	0	0	0	0	0	0	0	0
M7		0				13			0	0			0	0		17	0		0	0	0	0	0	0	0	0	0	1	0	0	0	0	0	0	0
M6		0				35			0	0			0	0		8	0		0	0	0	0	0	0	0	0	0	1	0	0	0	0	0	0	0
M5		0				40			2	39			18	10		8	0		26	0	0	14	0	0	0	0	3	21	8	0	1	1	3	0	0
M4		0				0			0	7			0	0		3	0		2	0	0	0	0	0	0	0	0	2	0	0	0	0	0	0	0
M3		0				0			0	6			0	0		1	0		0	0	0	0	0	0	0	0	0	0	0	0	0	0	0	0	0
FM		0				0			0	0			0	0		0	0		2	0	0	0	0	0	0	0	0	0	0	0	0	0	0	0	0
Hybrid		0				0			0	4			0	0		0	0		0	0	0	5	0	0	0	0	0	1	0	0	0	0	0	0	0
Fhybrid		0				0			0	1			2	0		0	0		2	0	0	5	0	0	0	0	0	1	0	0	0	0	0	0	0
HexNAc(3)(x)		0				0			0	0			4	1		0	0		12	0	0	15	0	0	0	0	0	19	0	0	2	0	0	0	0
HexNAc(3)(F)(x)		0				0			3	2			13	9		0	0		21	0	0	26	8	11	0	0	4	35	3	0	3	0	0	0	0
HexNAc(4)(x)		0				0			0	4			1	0		0	0		0	0	0	10	0	0	0	0	0	0	3	0	5	0	0	0	0
HexNAc(4)(F)(x)		0				0			57	6			53	44		0	35		29	23	84	19	41	72	0	9	15	8	64	8	60	47	6	0	0
HexNAc(5)(x)		0				0			0	1			0	0		0	0		0	0	0	0	0	0	0	0	0	0	0	0	1	5	0	0	0
HexNAc(5)(F)(x)		0				0			22	3			7	26		0	54		7	76	16	4	28	17	0	58	40	9	10	66	24	28	21	0	0
HexNAc(6+)(x)		0				0			0	0			0	0		0	0		0	0	0	0	0	0	0	0	0	0	0	0	0	0	0	0	0
HexNAc(6+)(F)(x)		0				0			0	0			0	1		0	0		0	0	0	0	13	0	0	32	10	0	0	26	1	16	21	0	39
Unoccupied		0				7			15	27			2	8		12	11		0	0	0	0	9	0	0	2	29	0	8	0	3	3	50	0	61
Core		0				0			0	2			0	0		2	0		0	1	0	1	0	0	0	0	0	1	4	0	0	0	0	0	0
Oligomannose						93			2	46			18	10		84	0		30	0	0	14	0	0		0	3	25	8	0	1	1	3		0
Hybrid		0				0			0	5			2	0		0	0		2	0	0	11	0	0	0	0	0	3	0	0	0	0	0	0	0
Complex		0				0			83	22			78	82		3	89		68	100	100	75	90	100	0	98	69	72	84	100	96	96	48	0	39
Unoccupied		0				7			15	27			2	8		12	11		0	0	0	0	9	0	0	2	29	0	8	0	3	3	50	0	61
Fucose		0				0			83	11			75	81		0	89		60	99	100	54	90	100	0	98	69	53	77	100	88	90	48	0	39
NeuAc		0				0			7	8			15	3		0	0		2	40	23	20	0	0	0	6	3	2	22	0	64	15	7	0	12
